# Accurate Analysis of Anisotropic Carrier Mobility and Structure–property Relationships in Organic BOXD Crystalline Materials

**DOI:** 10.3389/fchem.2021.775747

**Published:** 2021-11-11

**Authors:** Shi-Ping Wang, Yu Wang, Fang-Yi Chen, Hai-Tao Wang, Fu-Kit Sheong, Fu-Quan Bai, Hong-Xing Zhang

**Affiliations:** ^1^ International Joint Research Laboratory of Nano-Micro Architecture Chemistry, Laboratory of Theoretical and Computational Chemistry, Institute of Theoretical Chemistry and College of Chemistry, Jilin University, Changchun, China; ^2^ Key Laboratory of Automobile Materials (MOE), Institute of Materials Science and Engineering, Jilin University, Changchun, China; ^3^ Department of Chemistry and Institute for Advanced Study, Hong Kong University of Science and Technology, Kowloon, China; ^4^ Beijing National Laboratory for Molecular Sciences, Beijing, China

**Keywords:** charge mobility, crystal structure, BOXD derivatives, transfer integral, reorganization energy

## Abstract

Charge mobility is an essential factor of organic crystalline materials. Although many investigators have made important progress, the exact relationship between the crystal structure and carrier mobility remains to be clarified. Fortunately, a series of bis-1,3,4-oxadiazole derivatives have been successfully prepared and reported. They have similar main molecular fragments but different crystal packing modes, which provide an ideal research objective for studying the effect of molecular packing on charge mobility in organic photoelectric conversion systems. In this work, the charge mobilities of these molecules are systematically evaluated from the perspective of first-principles calculation, and the effect of a molecular overlap on orbital overlap integral and final charge carrier mobility is fully discussed. It can be seen that the small intermolecular distance (less than 6 Å) is the decisive factor to achieve high electron mobility in *π* stacking, and better mobility can be obtained by increasing the hole migration distance appropriately. A larger dihedral angle of anisotropy is an important point limiting the charge mobility in the herringbone arrangement. It is hoped that the correlation results between the crystal structure and mobility can assist the experimental study and provide an effective way to improve the photoelectric conversion efficiency of the organic semiconductor devices and multiple basis for multiscale material system characterization and material information.

## Introduction

Compared with inorganic materials, organic materials in our daily life have unique advantages in terms of price, ease of property, fine-tuning, and flexibility, etc. The structure of organic materials is complicated, and there are two types of organic materials in general: crystalline organic materials and amorphous organic materials. However, even for the simplest single crystalline materials, it is difficult to find a precise connection between the crystal structures and their micromechanisms such as anisotropic practical transport, structural deformation, and mechanical properties of anisotropy (Zhang et al., 2016; Ji et al., 2017; and Lin et al., 2020). There are enormous applications of organic crystals, such as OFET and OLET (Wise et al., 2018; Liu S. et al., 2020; Bi et al., 2021; and Wang et al., 2021). In particular, their requirements for crystal quality and high mobility have prompted the development of more crystal materials (Cho et al., 2020; Sharma et al., 2020; and Chen et al., 2021). Therefore, the relationship between the structure and charge mobility, especially the direction of mobility, becomes an important area of study. This is also an urgent issue that is to be solved by theoretical calculations (Chen et al., 2014; Chen et al., 2016; Yang et al., 2016; Rehn et al., 2018; and Chen X. et al., 2019).

In organic semiconductor crystals, non-covalent interactions such as hydrogen bonding, *π*–π stacking, CH–π, and anion–π^+^/cation–π− interactions play significant roles in many aspects (Wang et al., 2017). They not only have an important effect on controlling the molecular structure but also play a decisive role in the properties of materials (Ma et al., 2016; Benito-Hernández et al., 2018; Nowak-Król and Würthner, 2019; and Xing et al., 2021). In fact, much work has been carried out to evaluate the relationship between the crystalline structure and material properties. Among them, charge mobility is one of the most crucial factors of organic semiconductor crystals, as seen from the fact that the mobilities of organic semiconductor crystals have been greatly improved since it was first used in transistors. A great deal of experimental and theoretical research studies has focused on the improvement of the charge mobility (Zhang et al., 2013; He et al., 2018; Ozdemir et al., 2018; Chen et al., 2019b; and Liu Y. et al., 2020). By changing the length and position of the side chain of several kinds of semiconducting materials, Wang and Grozema et al. have modified the molecular packing pattern and thus changed the charge mobility (Grozema et al., 2002; Lei et al., 2013; and Wang et al., 2013). In addition, previous studies have also found that the induced charge transfer rate increases with the decrease in the *π*–π stacking distance between molecules. Anthory et al. have also found that controlling the general type of interaction present in the crystal (herringbone vs *π* stacking) can improve the transport performance of organic semiconductors (Anthony, 2006). But what is the exact factor that influences the transport ability when the crystal interaction is altered? A lot of research efforts have been put to identify the factor(s), and hopping barrier and carrier energy level had once been considered the causes (Fornari and Troisi, 2014). But Shuai et al. believe that the electronic coupling is the determining factor (Geng et al., 2019). Actually, high charge mobilities are exactly what researchers want, but although so many studies have been conducted, people still cannot come up with a specific relationship that connects the structures to their transport abilities. Here, we aim to search for the rule of change and influence regularities of their carrier transport properties in all directions to give a systematic explanation.

To achieve this goal, a series of symmetric alkoxy-substituted bis-1,3,4-oxadiazole derivatives (BOXD-o, BOXD-m, BOXD-p, BOXD-D, and BOXD-T) (CCDC numbers are 293679, 1448062, 1875779, and 1875781-1875783, respectively) has been chosen, which has been synthesized by Wang et al. (Chen et al., 2019a). The molecules only differ by their positions of substituents, but the crystal structures formed by these molecules are radically different, even though the molecular skeleton remains largely similar. Even BOXD-o has two kinds of crystal structure arrangement. Due to the fact that substituents tend to have little effect on the charge transfer properties of molecules, these molecules would serve as good materials to help us find the relationship between the crystal structures and their mobilities. Besides, previous researchers always use the Marcus theory to model the charge transfer process, but recent studies have found that this theory cannot fit the experimental results well. That is because the Marcus theory needs to guarantee both the adiabatic limit and the high-temperature limit $\left(k_{b}T\gg \hbar \omega \right)$ , but high-temperature approximation fails in the high-frequency vibration region. In order to consider the high-frequency vibration region as well, a quantum nuclear tunneling model presented by Shuai et al. is added to alleviate this problem in the current article, which is a good method to contain the influence of high- and low-frequency vibration (Nan et al., 2009; Geng et al., 2012; and Jiang et al., 2019). Because of its unique advantage in the high-frequency regime, it can better explain the reason underlying our key issue—how is carrier mobility affected by the way of stacked molecules.

**CHART 1:**
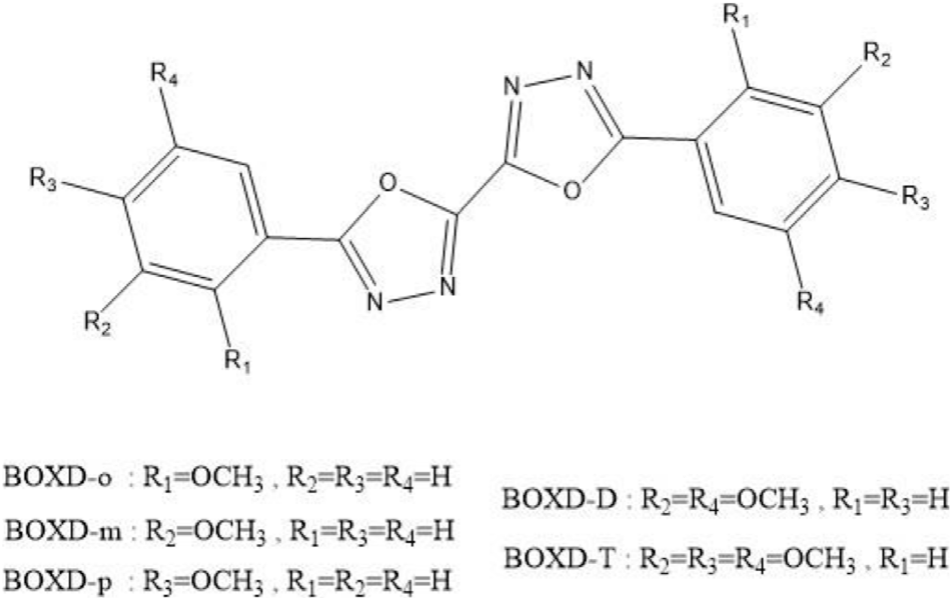
Molecular structures of alkoxy-substituted bis-1,3,4-oxadiazole derivatives.

Theoretical calculation of the electronic structure of organic semiconductor system is an effective means to analyze the micro-carrier mobility. In this article, we establish a causal relationship between charge mobilities and stacking modes by using theoretical simulation to parameterize the intrinsic properties of organic materials to have a full-dimensional and multi-faceted understanding. And based on these research studies, we find what kind of crystal structure is most conducive to charge transfer and explain the reason. We anticipate that after this study about more comprehensive material informatics, the interpretation of structure–activity relationships in organic crystal materials will be advanced and the design of new materials will be greatly promoted.

## Computational Details

Theoretical research on charge mobility is often based on the Marcus theory, but the high-frequency modes which may also play considerable role in charge mobility are neglected in this approach. In this study, the tunneling-enabled hopping models are employed to describe the charge transport, which has showed great advantages in the high-frequency regime. In this model, each hopping event is viewed as a non-adiabatic electron transfer reaction; the charge transfer (for electron) between two adjacent molecules follows the reaction M + M-→M- + M, where M is the molecule undergoing the electron transfer. The quantum mechanical charge transfer rate from one molecule to another under the perturbation theory and the displaced harmonic oscillator approximation can be derived from Fermi’s golden rule. The rule states that

$$W_{i\to f}=\frac{2\pi }{\hbar^{2}}{\left|\langle \Phi_{f}\left|H^{\prime }\right|\Phi_{i}\rangle \right|}^{2}\times \sum_{\upsilon }\sum_{\upsilon^{\prime }}P_{i\upsilon }{\left|\langle \Theta_{f\upsilon^{\prime }}|\Theta_{i\upsilon }\rangle \right|}^{2}\delta \left(\omega_{f\upsilon^{\prime },i\upsilon }\right),$$ (1)

where $\Phi_{i\left(f\right)}$ and $\Theta_{i\left(f\right)}$ represent the electronic and vibrational wave functions separately, and $P_{i\upsilon }$ denotes the Boltzmann distribution function given as follows:

$$P_{i\upsilon }={\left[\sum_{\upsilon }\exp\left(\frac{-E_{i\upsilon }}{kT}\right)\right]}^{-1}\exp\left(\frac{-E_{i\upsilon }}{kT}\right).$$ (2)

Under the displaced harmonic oscillator approximation (Lin et al., 2002; and Nan et al., 2009), Eq. 1 can be translated as follows:

$$k_{if}=\frac{{\left|V_{fi}\right|}^{2}}{\hbar }\int_{-\infty }^{\infty }dt\times \exp\{-i\omega_{fi}t-\sum_{j}S_{j}\left[\left(2{\bar{n}}_{j}+1\right)-{\bar{n}}_{j}e^{-i\omega_{j}t}-\left({\bar{n}}_{j}+1\right)e^{i\omega_{j}t}\right]\}$$ (3)

Here, $V_{fi}$ is the transfer integral between the final and initial states. For the self-exchange reaction, $\omega_{fi}=\Delta G^{0}/\hbar$ goes to zero, where $\Delta G^{0}$ is the Gibbs free energy difference between the final state and the initial state. $S_{j}=\lambda_{j}/\hbar \omega_{j}$ is the Huang–Rhys factor of the j -th normal mode, which is a measure of the electron–phonon coupling strength. $\omega_{j}$ and $\lambda_{j}$ are the vibrational frequency and reorganization energy of the vibrational mode j , respectively. ${\bar{n}}_{j}=1/\left(e^{\hbar \omega_{j}/k_{B}T}-1\right)$ is the phonon occupation number of the j -th normal mode. It should be noted that the convergence of the numerical integration of Eq. 3 can be achieved by choosing the vibrational mode with the largest Huang–Rhys factor and short-time approximation.

From Eq. 3, it can be seen that the electron transfer integral $V_{fi}$ , vibrational frequency $\omega_{j}$ , and the Huang–Rhys factor $S_{j}$ are the three important parameters to determine the charge transfer rate. The transfer integrals for the nearest-neighbor dimers along the transport pathways in bulk crystals can be calculated by using the site energy correction method given as follows:

$$V_{mn}=\frac{V_{mn}^{0}-1/2\left(e_{m}+e_{n}\right)S_{mn}}{1-s_{mn^{2}}}.$$ (4)

Here, $V_{mn}^{0}=\langle \Phi_{m}\left|H\right|\Phi_{n}\rangle$ , $e_{m\left(n\right)}=\langle \Phi_{m\left(n\right)}\left|H\right|\Phi_{n\left(m\right)}\rangle$ , and $S_{mn}=\langle \Phi_{m}\left|S\right|\Phi_{n}\rangle$ , where $\Phi_{m\left(n\right)}$ is the lowest unoccupied molecular orbital (for electron transport) or the highest occupied molecular orbital (for hole transport)of the isolated molecule in dimer. H and S are the dimer Hamiltonian and the overlap matrices, respectively. The sign of transfer integrals only indicates the combination type of the frontier orbital. The vibrational frequencies of the normal modes for the neutral and anion states (or cation states) associated with the electron (hole) transfer process are calculated using the Gaussian 16 program (Frisch et al., 2016) with suitable functionals, which are ωb97xd (Chai and Head-Gordon, 2008) and 6–31G (d,p) all-electron basis sets chosen from benchmarking. The Huang–Rhys factor $S_{j}$ and the reorganization energy $\lambda_{j}$ of every normal mode can be calculated using MOMAP program (Nan et al., 2009; Shuai et al., 2014a; Shuai et al., 2014b; and Niu et al., 2018). The orbital overlap is calculated using Multiwfn program (Lu and Chen, 2012). Generally speaking, reorganization energy has contributions from both internal reorganization energy $\lambda_{int}$ and external reorganization energy $\lambda_{ext}$ . The former reflects the geometric relaxation of individual molecules upon going from the neutral (Shuai et al., 2014b) to the charged state, and vice versa. And the latter reflects the electronic polarization of the surrounding molecules, which is usually neglected. The internal reorganization energy is dominant and can be evaluated either from the adiabatic potential energy surfaces or from the normal-mode analysis. The partition of the internal reorganization energy into the contributions from each vibrational mode is given as follows:

$$\lambda_{\mathrm{int}}=\sum \lambda_{i}=\sum \hbar \omega_{i}S_{i}=\sum \frac{1}{2}\omega_{i}^{2}\Delta Q_{i}^{2}.$$ (5)

Here, $\Delta Q_{i}$ represents the displacement along the normal mode i between the equilibrium geometries of the neutral and charged molecules. Within the hopping model, the charge mobility can be expressed by the following Einstein relation equation:

$$\mu =\frac{e}{k_{B}T}D,$$ (6)

The diffusion coefficient D is calculated by the kinetic Monte Carlo simulation approach. One molecule has been arbitrarily chosen as the initial charge center. The charge is only allowed to hop to the nearest neighbor molecules. The hopping probability is evaluated as $P_{\alpha }=k_{mn}/\sum_{n^{\prime }}k_{mn^{\prime }}$ , where $k_{mn}$ is the hopping rate with nuclear tunneling from site m to n , and n′ represents all nearest molecules. The residence time of the carrier at site m is $1/\sum_{n^{\prime }}k_{mn^{\prime }}$ . At each step, a random number is uniformly generated between 0 and 1. If $\sum_{\alpha =1}^{n-1}P_{\alpha }<r\leq \sum_{\alpha =1}^{n}P_{\alpha }$ , the hopping distance is taken as the intermolecular centroid distance. The charge then goes to the neighbor in the α -th direction as the nearest position of the charge. Finally, the carrier mobility can be obtained from the Einstein relation equation.

## Results and Discussions

### Crystal Structure Analysis

Many unique physical properties of organic crystalline materials are resulted from their long ranged order—periodic structure. Because of its well-defined molecular structure, molecular packing, and intermolecular interaction, single crystal is considered as an ideal model to investigate the relationship between the properties and structures. To conveniently and intuitively describe the spatial relationship between the reference molecule and its neighbors in single crystal, we have divided the neighbors into *π* stacking and herringbone arrangement and then discussed them separately. Firstly, to describe the *π* stacking of this series of BOXD complexes, slip distance between the nearest adjacent molecules along the direction of x, y, and z is needed. The molecular long axis (y) is defined as a line through the two para-C in terminal benzene rings; in the meanwhile, another line in the molecular plane that is also perpendicular to the molecular long axis can be defined as the molecular short axis (x); besides, the *π* stacking direction (z) is perpendicular to the x-y plane. Although the BOXD-D crystal is full of *π* stacking arrangements, herringbone arrangement also exist in the structure of other BOXD derivatives which cannot be simply defined by the slip distance. In this case, the angle between two molecular long axes (σ) and dihedral angle between two molecular planes (φ) are used to describe the relative directions of primary herringbone arrangement. In addition, the intermolecular distance is taken as the distance between two molecular centers.

**FIGURE 1:**
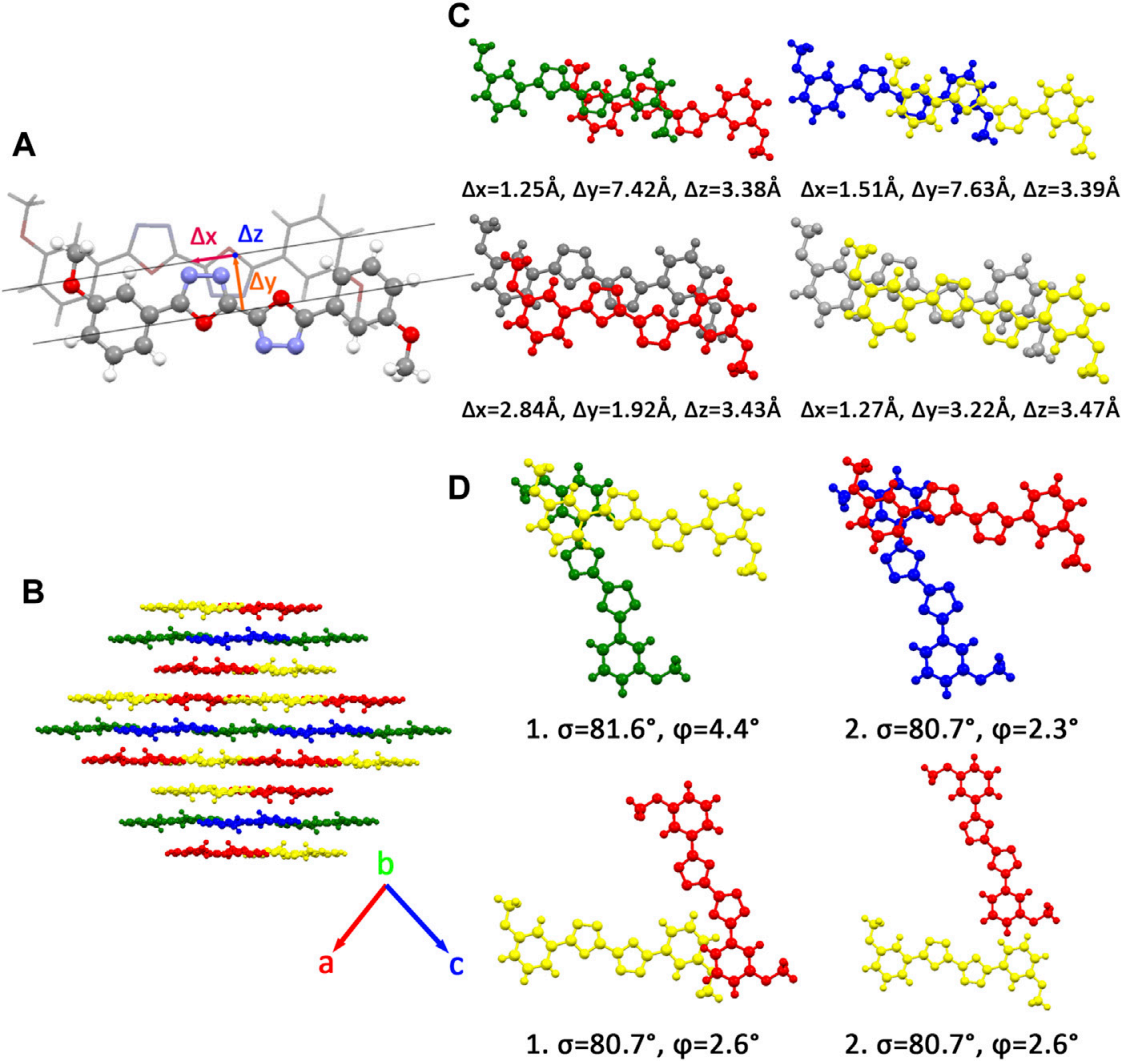
**(A)** Molecular structure of single molecular **BOXD-m**. Δx and Δy represent the slip distance along the short and long axes, Δz is the *π*–π distance. **(B)** Crystal structure and the crystal cell directions of **BOXD-m**. **(C)** Primary relative positions of the bimolecular in *π* stacking. (The gray molecule has the same color with another molecule in the pairs; we use gray color to better indicate the relative location.) **(D)** Primary relative positions of the bimolecular in herringbone arrangement with the angle between two long axes (σ) and dihedral angle between molecular planes (φ) .

There are four kinds of molecular structures with distinct torsion angles in a BOXD-m crystal. BOXD-m also exhibits layered assembly structure features, and each molecular layer has two kinds of molecules. Molecule 1 (green) and molecule 2 (blue) are in the same molecular layer (a), while molecule 3 (red) and molecule 4 (yellow) are in another layer (b). The layers are arranged in the a-b-b manner with intermolecular *π*–π interactions in between. As present in Figure 1, significant slipping is present between 1–3 dimers (green and red pairs in Figure 1) and 3–3 dimers (red and gray pairs in Figure 1). In 1–3 dimers, the slip distance along the molecular long axes is about 7.42 Å (Δy); the slip distance between the molecular short axes is about 1.25 Å (Δx); and the contact distances are in a value of 3.38 Å (Δz). In 3–3 dimers, a larger slip distance along the molecular short axes and a smaller slip distance along the molecular long axes could be observed (Δx = 2.84 Å and Δy = 1.92 Å) than those in 1–3 dimers, but the distance of intermolecular *π*–π interactions is similar at 3.43 Å (Δz). Molecule 2 and molecule 4 stack with similar displacement along the molecular long and short axes are shown in Figure 1. At the same time, herringbone arrangement also can be found. There are two kinds of primary relative directions for the arrangements between the a and b layers: for molecular 1 and molecular 4, the angle between long axes is 81.6° (σ) and the dihedral angle between two molecule planes is 4.4° (φ) , but between molecular 2 and molecular 3, the long axis angle and the dihedral angle slightly changed and became 80.7° (σ) and 2.3° (φ) . For the b–b layers, there are also two kinds of primary relative directions, but the angle between the long axes and dihedral angle of two molecule planes are identical, which are 81.6° (σ) and 4.4° (φ) , respectively.

**FIGURE 2:**
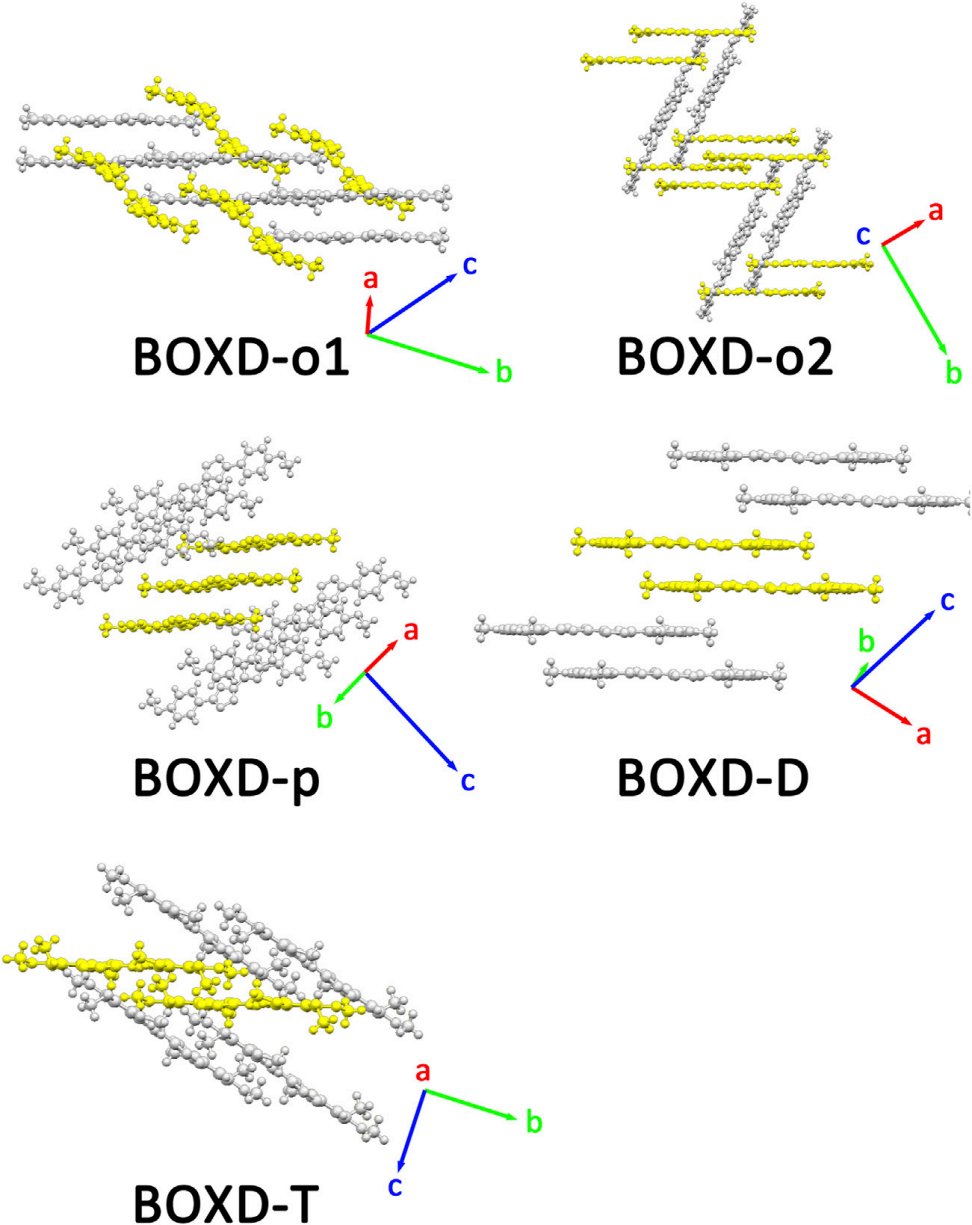
Crystal structures and crystal cell directions of **BOXD-o1**, **BOXD-o2**, **BOXD-p**, **BOXD-D**, and **BOXD-T**.

Two crystal structures of BOXD-o have been obtained so far (Figure 2). The *π* stacking slip distance between BOXD-o-1 along the long axis (Δy) is 0.40 Å, distance along the short axis is 1.72 Å (Δx), and the distance from *π*–π interactions is 3.52 Å (Δz). In this crystal structure, herringbone arrangement also can be found in different layers. The distance from the nearest adjacent molecules is 10.64 Å, the long axis of these two molecules is nearly perpendicular to each other which is 89.5°, and the dihedral angle of two molecular planes is 27.6° (Figure S1). Molecules in BOXD-o-2 show different *π* stacking with a contact distance of 3.37 Å (Δz), and displacement of the nearest molecules in *π* stacking along the molecular long axis is much longer than it is in BOXD-o-1, which is 5.75 Å (Δy), and the slip distance along the molecular short axis is about 0.81 Å (Δx). The long axis angle of primary herringbone arrangement is 57.2°, and the dihedral angle of two molecule planes is about 61.7°, with a distance of two molecules being 9.32 Å (Figure S2) Molecules in BOXD-p exhibit a planar molecular structure, with the existence of both *π* stacking and herringbone arrangement. In the *π* stacking, the slipping distance along the molecular long axis and short axis of the nearest adjacent molecules is about 5.37 Å (Δy) and 1.11 Å (Δx), respectively, and the contact distance is at 3.38 Å (Δz). In the herringbone arrangement, the long axis angle is about 35.6°, which is much smaller than other packing modes, but the dihedral angle is as large as 67.8° than other molecules (Figure S3). There is no herringbone arrangement in the BOXD-D crystal, the slip distance of the nearest adjacent molecules in *π* stacking along the molecular long axis (Δy) and short axis (Δx), and *π*–π contact distances (Δz) are measured as 5.45 Å, 0.67 Å, and 3.32 Å (Δz), respectively. BOXD-D features a layered assembly structure (Figure S4). The slip distance of BOXD-T1 molecules along the molecular long axis and short axis is 5.15 Å (Δy) and 6.02 Å (Δx), respectively. This molecule can be considered as a special *π* stacking, but the distance of the nearest adjacent molecules is too large so that there is no overlap between the molecules. The *π*–π interaction distance is calculated as 2.97 Å (Δz). As for the primary herringbone arrangement, the long axis angle is 75.0° and the dihedral angle is 22.5° with a 5.7 Å intermolecular distance (Figure S5). Taking all the crystal structures together, the total distances in *π* stacking are between 4.5Å and 8.5Å, and it will become much larger from 5.7Å to 10.8Å in the herringbone arrangement. The long axis angles are at least 57°, except that in BOXD-p, it is as small as 35.7°. There are also various dihedral angles between molecule planes; among them, the molecules in BOXD-m are almost parallel to each other (Table 1).

**TABLE 1:** Crystal structure data of BOXD-o1, BOXD-o2, BOXD-p, BOXD-D, and BOXD-T.

π stacking	m-p1	m-p2	m-p5	m-p6	o1-p1	o2-p3	p-p1	D-p1	D-p2	T-p2
Total distance	4.91	8.41	4.86	8.32	3.94	6.71	6.42	6.41	13.28	8.46
Δx	1.27	1.51	2.84	1.25	1.72	0.81	1.11	0.67	0.32	6.02
Δy	3.22	7.63	1.92	7.42	0.40	5.75	5.37	5.44	12.7	5.15
Δz	3.47	3.39	3.43	3.38	3.52	3.37	3.38	3.32	3.52	2.97
**Herringbone arrangemen**	**D**	**m-p3**	**m-p4**	**m-p7**	**m-p8**	**o1-p2**	**o2-p1**	**o2-p2**	**p**	**T-p1**
Total distance	-	6.16	10.76	6.17	9.16	10.64	9.31	9.32	9.11	5.70
Σ	-	81.6	80.7	80.7	80.7	89.5	57.2	57.2	35.6	75.0
φ	-	4.4	2.6	2.3	2.6	27.6	61.7	61.7	67.8	22.5

### Electron Mobility Analysis

The ability for the series of BOXD derivatives to form a wide variety of single crystals simply by fine-tuning its substituents makes it an exceptional model for deep investigation of carrier mobility. This section will begin with the structural diversity of the previous section and emphasizes on the diversity of the charge transfer process.

**FIGURE 3:**
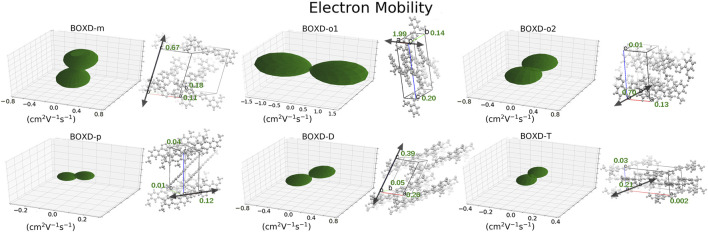
Three-dimensional images of electron mobility in six crystal structures. The mobilities of each direction are next to the crystal cell directions.

A comprehensive computation based on the quantum nuclear tunneling model has been carried out to study the charge transport property. The charge transfer rates of the aforementioned six kinds of crystals have been calculated, and the 3D angular resolution anisotropic electron mobility is presented in Figure 3. BOXD-o-1 has the highest electron mobility, which is 1.99 cm^2^V^−1^s^−1^, and the average electron mobility is also as large as 0.77 cm^2^V^−1^s^−1^, while BOXD-p has the smallest average electron mobility, only 5.63 × 10^-2^ cm^2^V^−1^s^−1^, which is just a tenth of the former. BOXD-m and BOXD-o-2 also have comparable electron mobility. Besides, all these crystals have relatively good anisotropy. Among them, the worst anisotropy appears in BOXD-m which also has the least ordered arrangement.

**FIGURE 4:**
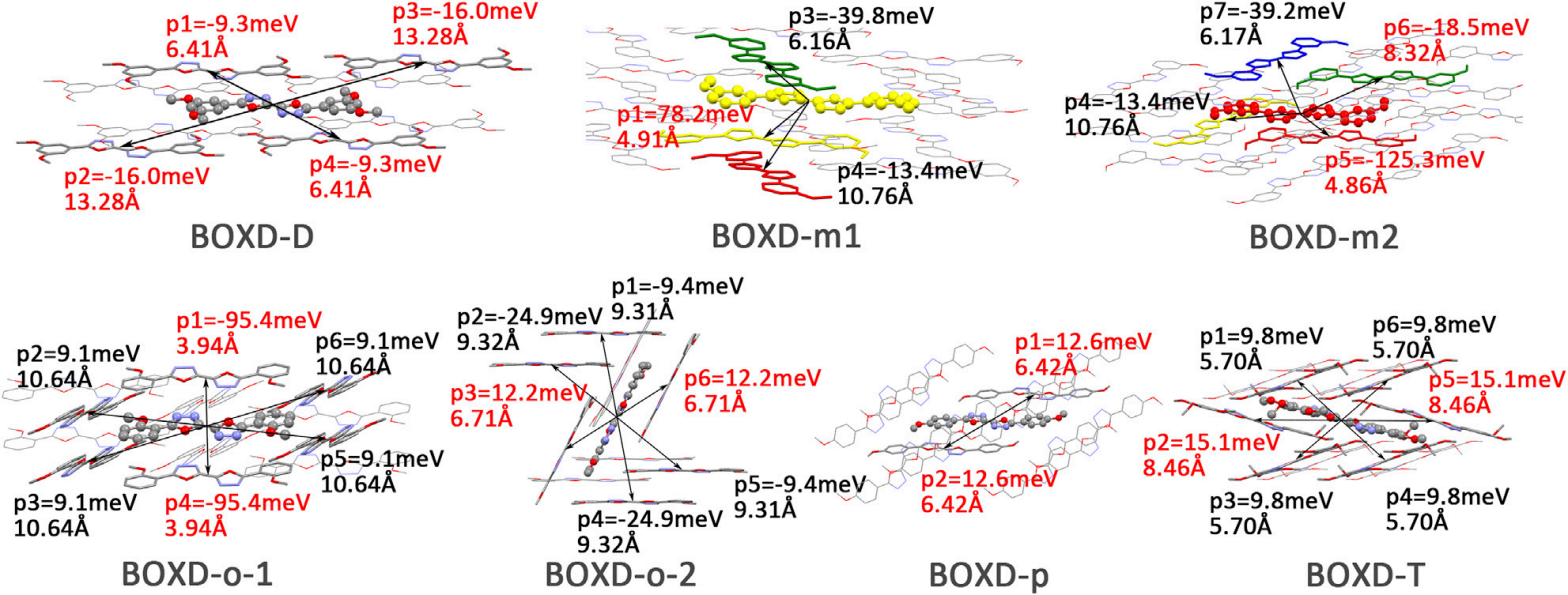
Transfer integral and intermolecular distance of primary electron transfer paths in each crystal structure. BOXD-m1 and BOXD-m2 need to be distinguished due to the complexity of intermolecular position; the molecular color is based on Figure 1. The transfer integral and intermolecular distance of *π* stacking are depicted in red, and herringbone arrangement are depicted in black.

Changing the position and number of substituents would affect electron mobility in different aspects, and here, the possible change in reorganization energy is first examined. The reorganization energies between anion and neutral molecules of these compounds have been analyzed (Figure S6). It can be noticed that the overall reorganization energies of these molecules are similar, and the normal modes corresponding to the highest reorganization energies are all contributed by the vibrations of two central-C. From the equation (Eq. 3), the difference in charge mobility is mainly related to the reorganization energy and transfer integral. If the influence in terms of structure reorganization is relatively small, transfer integral should be the critical factor of the mobility changes Figure 4 shows the primary transfer integral and intermolecular distance of these crystal structures. In fact, electron mobility is directly proportional to the transfer integral coupling between a molecule and its surroundings. But BOXD-m does not strictly follow this rule; they have the highest transfer integral but not the highest electron mobility. The reason is that although the transfer integrals are extraordinarily high in path 2 (b-b layer) and path 6 (b-b layer), which will truly cause larger mobility; transfer integrals are actually much lower in path1, path4, and path7 (a-b layer), and so the overall electron mobility will be limited when the electrons transfer through the a-b-b layer.

**FIGURE 5:**
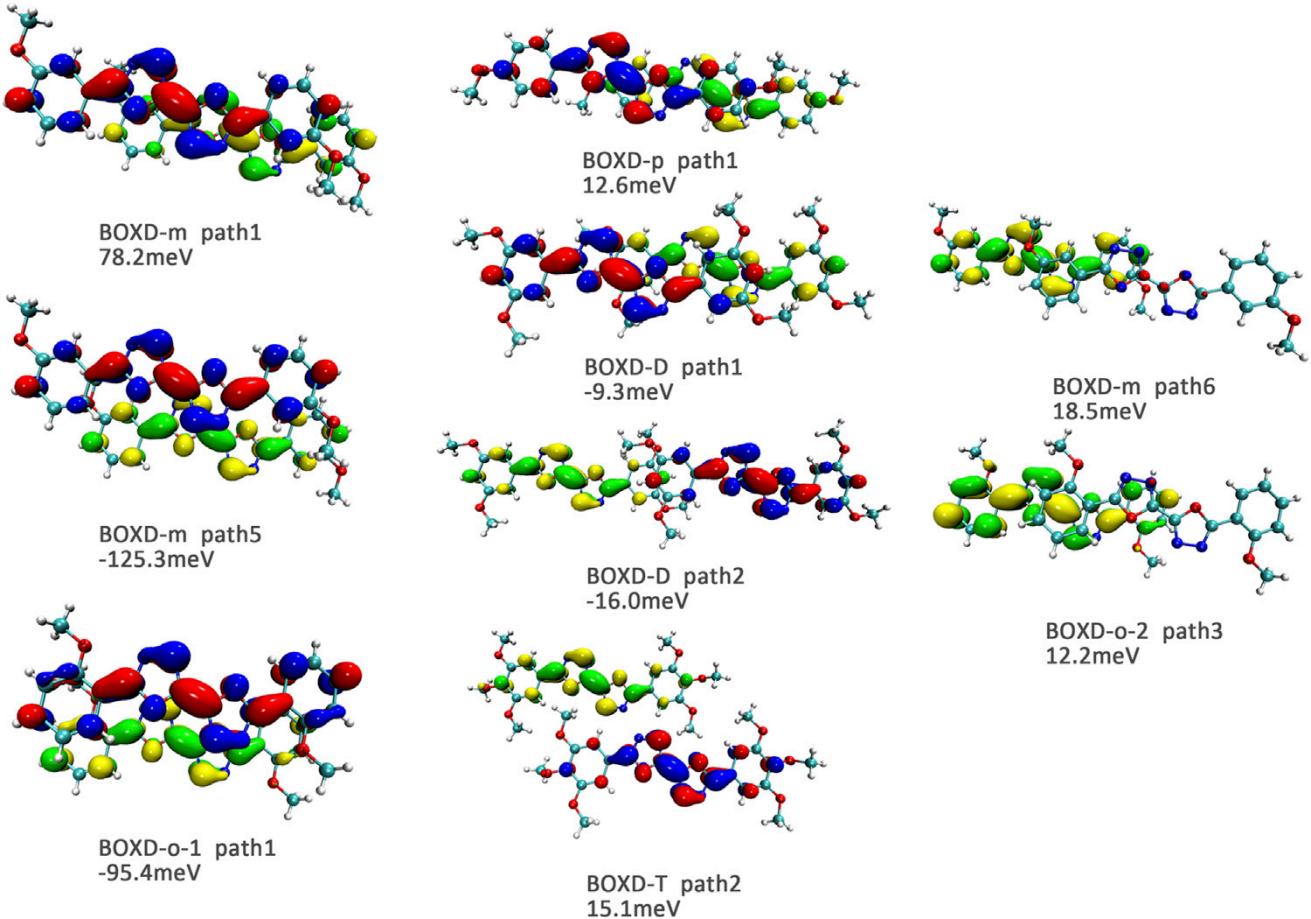
Bimolecular orbital distribution of primary electron transfer paths in *π* stacking. The positive phase is depicted in red and yellow, and the negative phase is depicted in blue and green.

Here, one should notice that the difference in the transfer integral of electron transfer can be explained through the intermolecular direction and the molecular orbitals, and it needs to be discussed separately for *π* stacking and herringbone arrangement. Here, the main charge transfer pathways were discovered and illustrated with Figure 5. For *π* stacking, there are basically three factors that combine into the final outcome: the Coulomb coupling, the nature of the overlapping orbitals, and the magnitude of slip distances. The positive Coulomb coupling value would make LUMOs distributed on both molecules when there are small slip distances. Close examination of path 1 and path 5 of BOXD-m and path 1 of BOXD-o-1 reveals that the bonding orbital overlaps with the bonding orbital and the antibonding orbital also overlaps with the antibonding orbital. The small slip distance of long axes (y) allows the molecular orbitals to couple strongly to each other. Under this circumstance, greater overlap and stronger coupling will result in larger transfer integral. On the other hand, if the bonding orbitals overlap with the antibonding orbitals because of the intermolecular slippage like path 2 of BOXD-D and path 2 of BOXD-T, the transfer integrals will be greatly reduced, even smaller than path 1 of BOXD-p and path 1 of BOXD-D with much less overlap. The other distribution mode is that the LUMOs are located on one of the two molecules because the Coulomb coupling value is negative. With this distribution, the electron transport between the two molecules becomes more difficult. In path 7 of BOXD-m and path 3 of BOXD-o-2, the transfer integral is going to be small without the overlap between the molecular orbitals.

**FIGURE 6:**
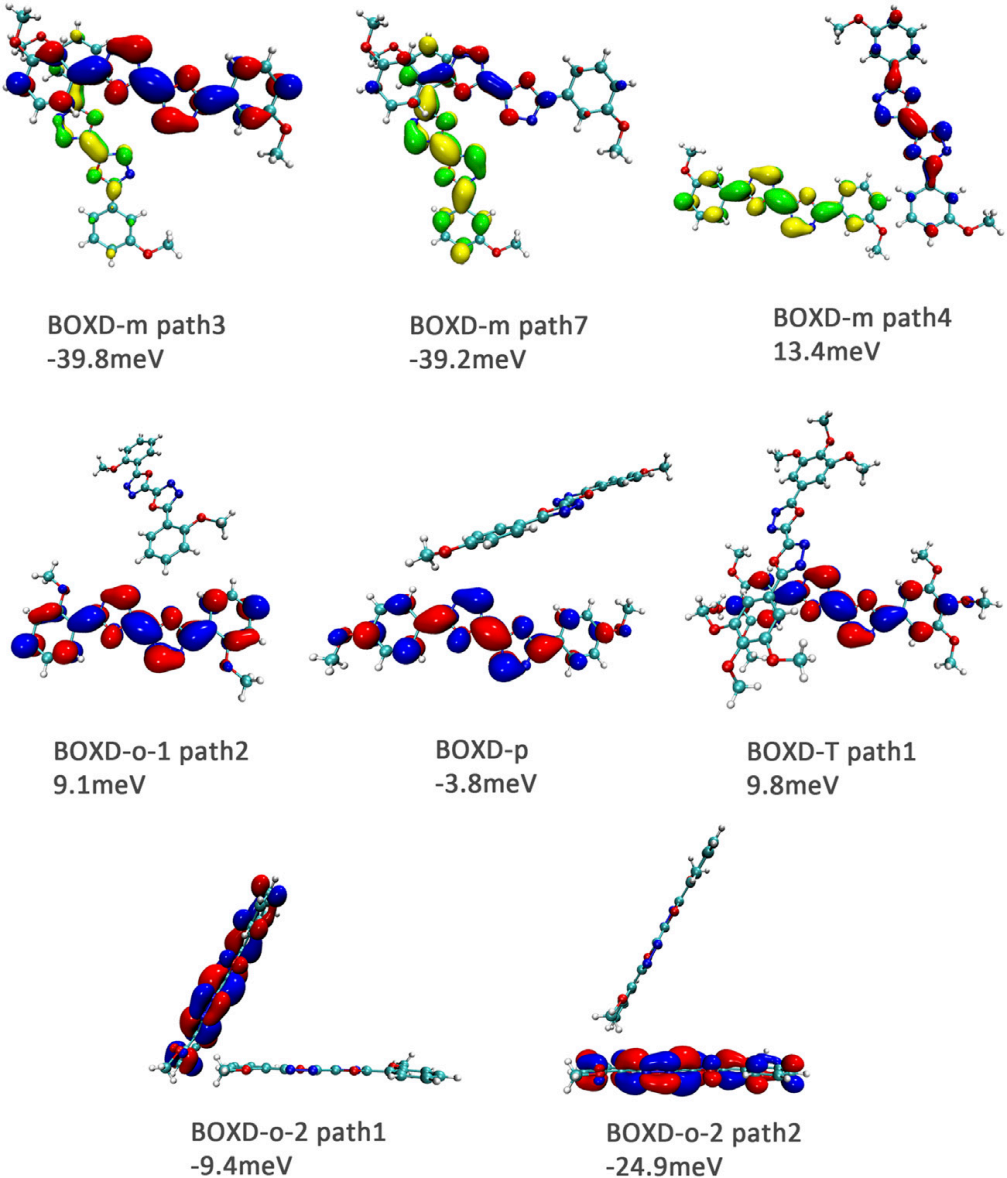
Bimolecular orbital distribution of primary electron transfer paths in herringbone arrangement. The positive phase is depicted in red and yellow, and the negative phase is depicted in blue and green.

It can also be noticed that the transfer integrals of herringbone arrangement are lower than those in *π* stacking (Figure 6). Taking a closer look into the LUMOs, it will be clear that without a positive Coulomb coupling value, the electrons are more or less concentrated on only one molecule, which will decrease the transfer integral for the reason mentioned before. The magnitude of the transfer integral is related to the overlap between the molecules and the dihedral angle between them. By comparing the different paths of BOXD-m, it can be found that the included angle and dihedral angle between the molecules of the two transport paths are basically same. But the transfer integrals will be larger with more molecular overlaps. Comparing the herringbone arrangement transport pathways of different molecules with an increase in the dihedral angle between molecules, it can be seen that the transfer integral decreases significantly. It can be considered that increasing the dihedral angle is another way to reduce the overlap. On the other hand, intermolecular distance does not seem to be a crucial factor of herringbone arrangement. By paying attention to path 2 of BOXD-o-1 and path 1 of BOXD-T, it can be found that they have similar angles and dihedral angles, but even though the distances between the molecules are very different, the change in the transfer integral is still not obvious. However, BOXD-o-2 does not fit into the aforementioned pattern, the transfer integral of path 3 is more than two times that of path 2, but the differences in the orbital overlap of these two paths cannot be visually observed. In this case, more specific calculation is needed to get a deeper understanding of this result. After the calculation of the overlap of the molecular orbitals, it can still be noticed that the molecular orbital overlap integral which will be mentioned in the next paragraph of path 2 is 2.6 times that of path 1, even the difference of the orbital overlap cannot be observed visually. It is also the overlap that leads to the difference of the transfer integral.

In order to obtain more information about the transfer integral, a deeper insight of interaction of the LUMO should also be evaluated. The *π*–π interactions are most obvious interaction, and n–π interactions can also be considered. In addition, the location of interactions, that is, the orbital overlap direction is shown in Figure S7 and Figure S8. After that, the intensity of interaction which can be evaluated by the orbital overlap integral is also depicted in Table 2. The transfer integrals of the primary transfer pathway are also given in Table 2, and they have already been sorted from the largest to smallest both in *π* stacking and herringbone arrangement. Attention to the orbital overlap integral, the similar tendency can also be found, except p–p1, o2–p3, and o2–p1. These three pathways have lower transfer integral, although they can achieve higher orbital overlap integral than others. A larger molecular overlap can be found in these pathways than the paths of D–p2 and T–p2. In this case, large orbital overlap integral is essential for electron transfer. As orbital overlap integral is reduced to orders of 10^−3^, the molecular overlap begins to show its negative effects to limit electron transfer.

**TABLE 2:** Transfer integral and orbital overlap integral of primary electron pathways in *π* stacking and herringbone arrangement.

π stacking	m-p5	o1-p1	m-p1	m-p6	D-p2	T-p2	p-p1	o2-p3	D-p1
Transfer integral	125.3	95.4	78.2	18.5	16.0	15.1	12.6	12.2	9.3
Orbital overlap	1.50E-02	1.34E-02	7.15E-03	4.10E-03	1.34E-03	1.51E-03	3.33E-03	2.80E-03	1.48E-04
**Herringbone arrangement**	**m-p3**	**m-p7**	**o2-p2**	**m-p4**	**T-p1**	**o2-p1**	**o1-p2**	**P**	**-**
Transfer integral	39.8	39.2	24.9	13.4	9.8	9.4	9.1	3.8	-
Orbital overlap	4.82E-03	4.73E-03	2.45E-03	1.31E-03	8.12E-04	8.52E-04	6.18E-04	3.36E-04	-

### Hole Mobility Analysis

**FIGURE 7:**
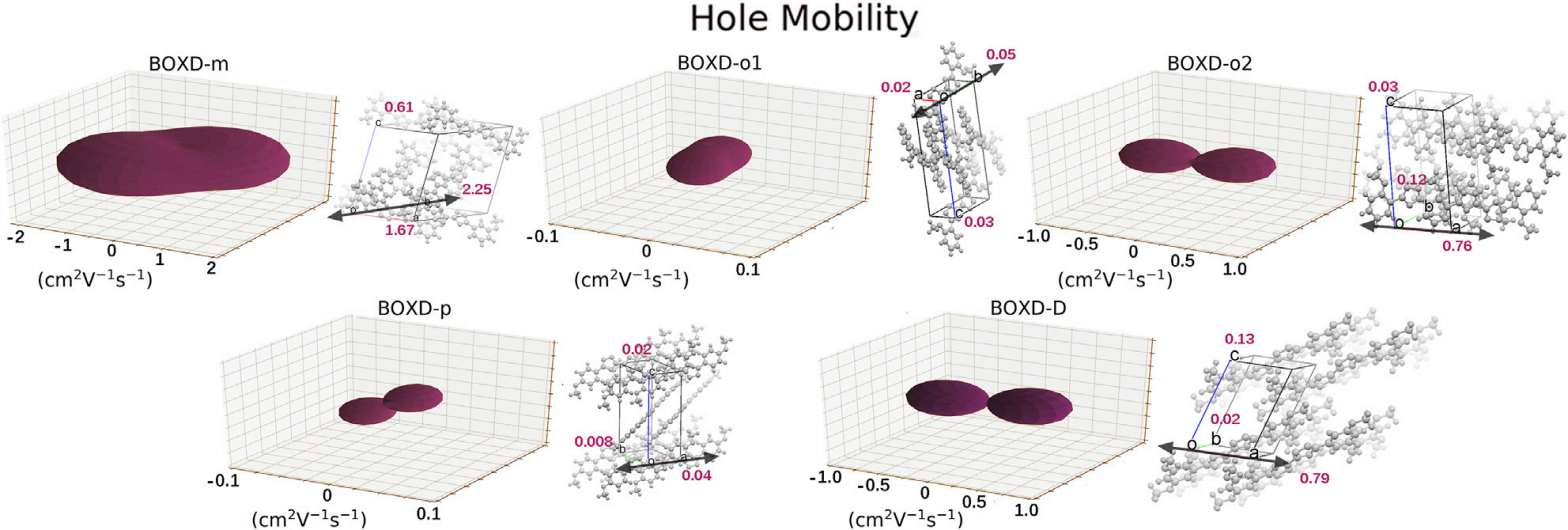
Three-dimensional images of hole mobility in six crystal structures. The hole mobility of BOXD-T is 0 and is not showed here. The mobilities of each direction are next to the crystal cell directions.

A dramatic difference can be found in the calculation of hole transfer in contrast to electron transfer. The largest mobility in hole transport can even achieve 2.15 cm^2^V^−1^s^−1^, which is contributed by BOXD-m with the largest average mobility of 1.43 cm^2^V^−1^s^−1^. After that, BOXD-D and BOXD-o2 also have good performance, and their average mobilities are 0.28 cm^2^V^−1^s^−1^ and 0.34 cm^2^V^−1^s^−1^, respectively. The average mobility of BOXD-o1 and BOXD-p are relatively small—3.55 × 10^-2^ cm^2^V^−1^s^−1^ and 2.39 × 10^-2^ cm^2^V^−1^s^−1^. The calculated mobility of BOXD-T is 0 cm^2^V^−1^s^−1^, it seems like this structure cannot be used as a hole transfer material. But as shown in Figure 7, the anisotropy of hole mobilities is not as good as that in electron transfer. The anisotropy of BOXD-m is not good, although it has the best mobility. Quite large hole mobility can also be found perpendicular to the direction of maximum transport, while the mobility of BOXD-o1 is almost the same for all directions.

**FIGURE 8:**
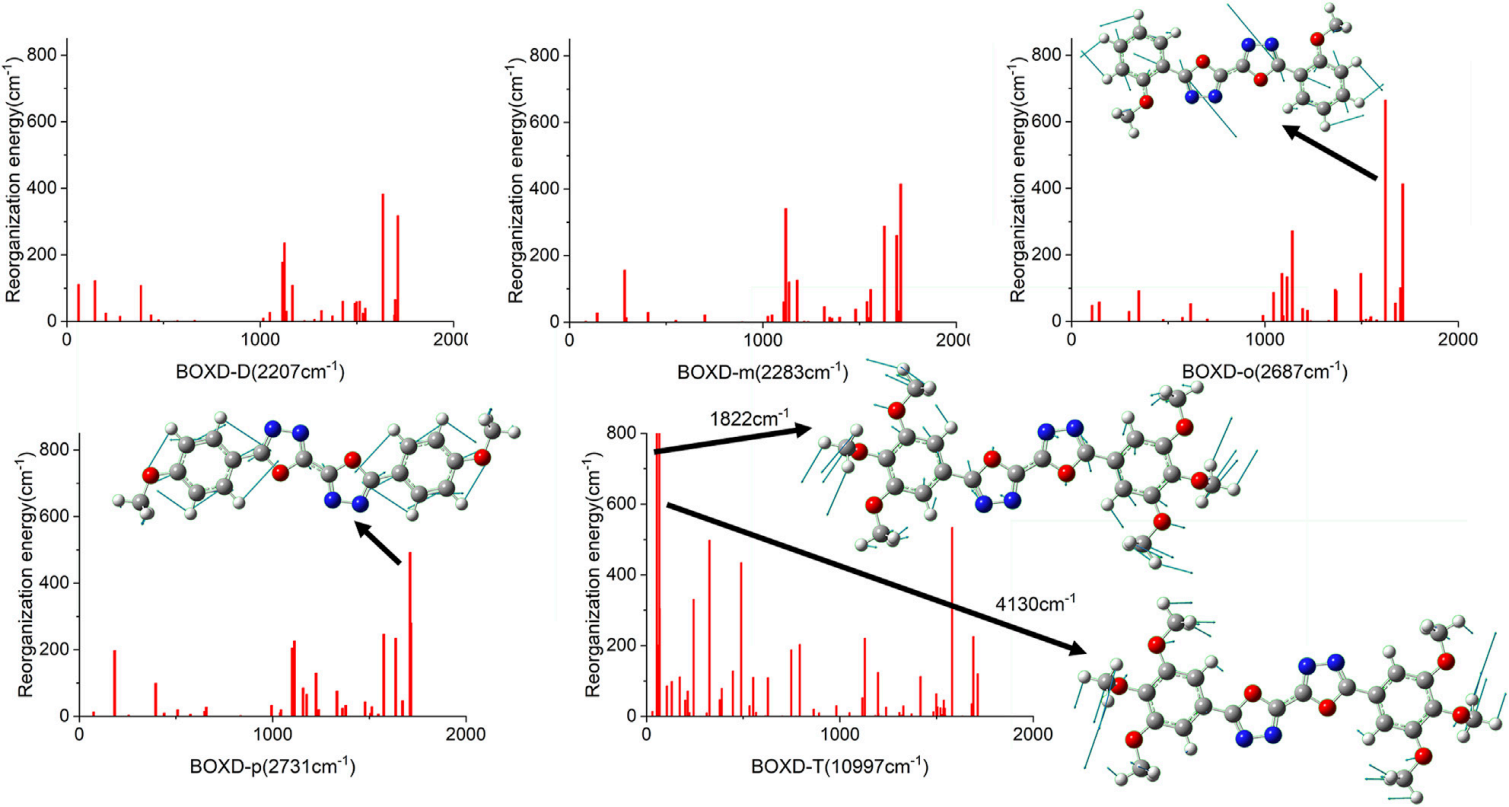
Calculated reorganization energies versus the normal-mode frequencies and the normal mode displacement vectors for the largest reorganization energy of five molecular structures.

Just like the analysis on the electron mobility, reorganization energy and the transfer integral should be considered in hole transport. In order to make clear the relationship between reorganization energies and normal-mode frequencies, their correspondences are plotted in Figure 8. The condition of the reorganization energies in hole transfer has great difference compared with that in electron transfer. When the methoxy group is attached to the meta-carbon of the benzene ring as in BOXD-D and BOXD-m, the minimum reorganization energy can be obtained. With the methoxide group changing its position to ortho and para carbon, reorganization energy will be higher in high frequency (1,000–2,000 nm). The first two highest peaks of reorganization energy in the high-frequency region are picked out, and then the two corresponding frequencies are decomposed into internal coordinates. The vibrations are contributed by the bis-1,3,4-oxadiazole molecular skeleton and are in the molecular plane. It also can be seen that the reorganization energies of BOXD-T are much larger than those for the others, especially for the low-frequency region (0–1,000 nm), suggesting a greater degree of structure distortion. The highest two vibration modes in low-frequency are both contributed by the methoxyl groups located at the terminal of the molecule vibrating into the molecular plane. Reorganization energy comes from geometry relaxation, which is related to the change of electron distribution.

**FIGURE 9:**
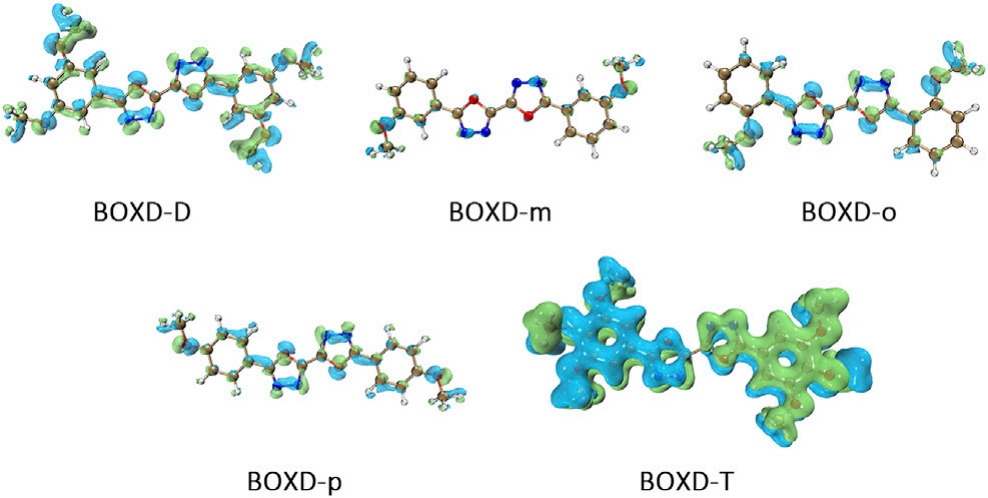
Electron density differences of neutral molecule and cation.

To achieve a better understanding of the difference in reorganization energy, it should be a good choice to examine the difference of electron density. Figure 9 shows the electron density difference between the neutral molecule and cation. It can be noticed that the difference in BOXD-m is tremendously small and much larger in BOXD-D, but the increases in position and degree (green) and the decreases in position and degree (blue) of electron density in BOXD-D are basically the same, which makes the reorganization energy of them smaller than others. While in BOXD-o and BOXD-p, the degree of electron density at the 5-member ring position is decreased, which causes more vibrational relaxation in the molecular skeleton. As for molecular BOXD-T, greater difference of electron density can be found at the position of the methoxyl-groups. In the process of losing electrons, not only the positively charged regions are more concentrated to the five-member ring but also electron rearrangement at the position of methoxyl group of para-C. In this case, the changes of the electron distribution will cause the structure of the methoxide group to be unstable and increase the vibration in the low-frequency region.

**FIGURE 10:**
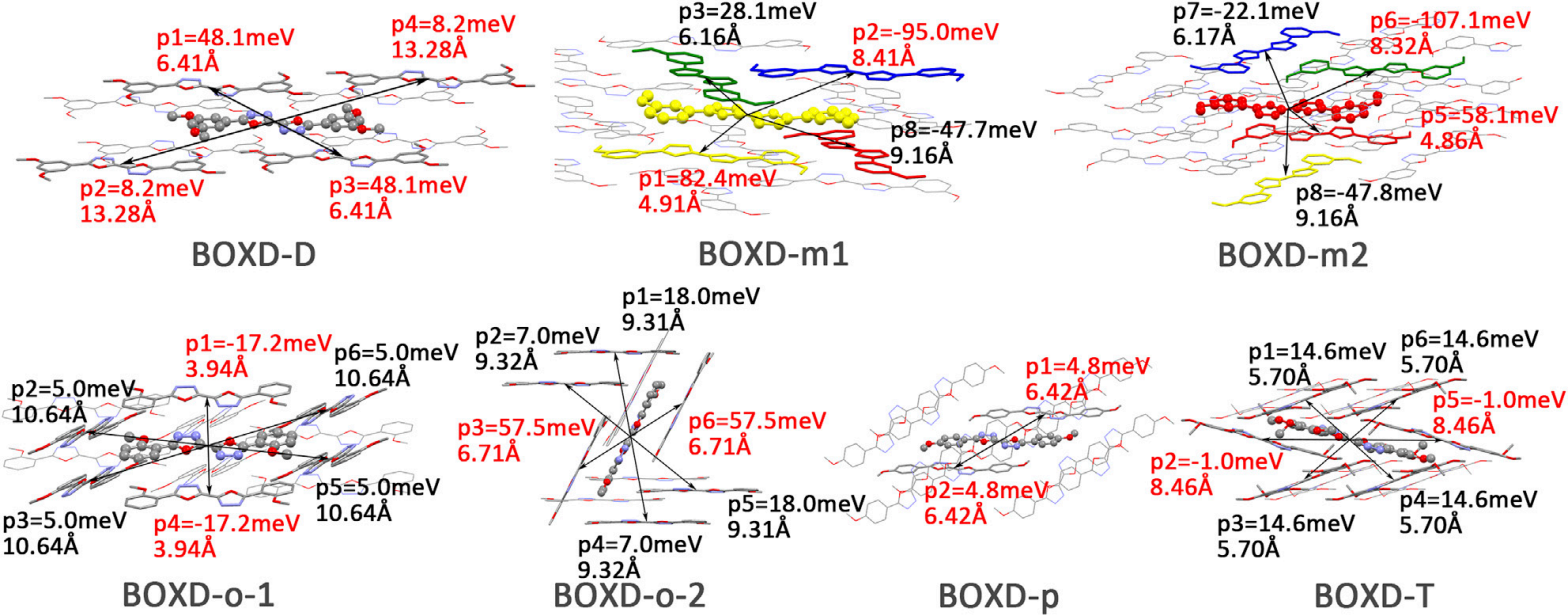
Transfer integral and intermolecular distance of primary hole transfer paths in each crystal structure. BOXD-m1 and BOXD-m2 need to be distinguished due to the complexity of intermolecular position, and the molecular color is based on Figure 1. The transfer integral and intermolecular distance of *π* stacking are depicted in red, and herringbone arrangement are depicted in black.

**FIGURE 11:**
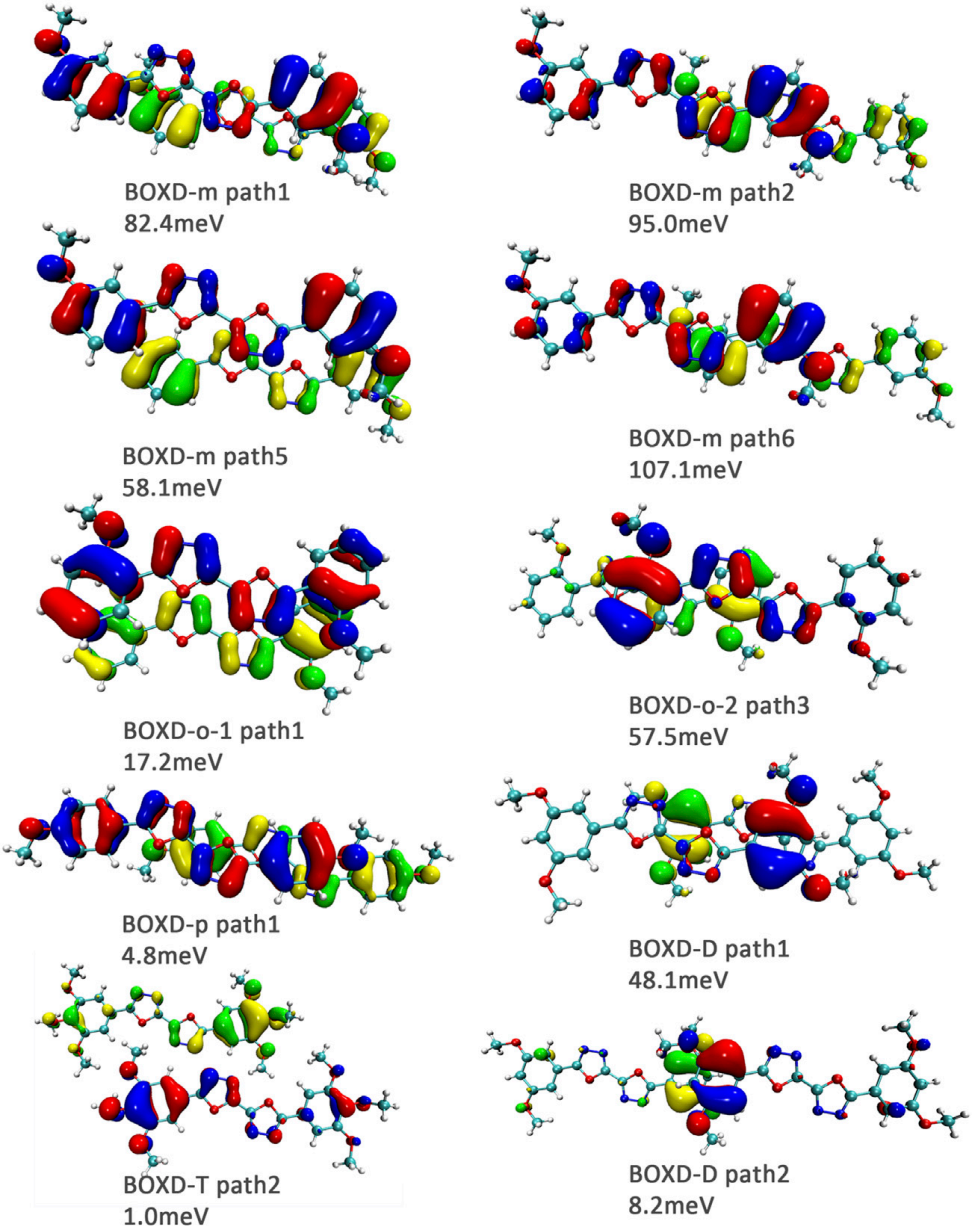
Bimolecular orbital distribution of primary hole transfer paths in *π* stacking. The positive phase is depicted in red and yellow, and the negative phase is depicted in blue and green.

Another crucial factor—transfer integral also have drastic difference compared to that in electron mobility. The transfer integral and the intermolecular distance of the primary hole transport path is also shown in Figure 10. The frontier molecular orbital is necessary for the analysis of transfer integral, but the analysis of hole transfer integral is dependent on the HOMOs (Figure 11). In *π* stacking, there are two general kinds of intermolecular stacking, the one is that the HOMO orbital of each monomer is still evenly distributed within the monomer, which may usually be associated with a small long-axis slip distance. In this case, the overlap of the HOMOs is proportional to the transfer integral between the molecules; the greater the overlap the molecular orbital overlap makes, electrons are more likely to be transferred between molecules, which can explain the change in transfer integrals. The path 1 of BOXD-m has the largest overlap and also the largest transfer integral, and as the overlaps decrease in the path 5 of BOXD-m and path 1 of BOXD-o-1, the transfer integral decreases accordingly. The transfer integral of BOXD-p will be smaller because of the larger molecular slip distance along with the smaller orbital overlap. In BOXD-T, there is no overlap in molecules; thus, the transfer integral is almost 0. The other stacking way is usually accompanied by large long-axis distance, which will concentrate the molecular orbitals on the overlapping part of the two molecules. It would be advantageous to separate the electron and the hole as it would also be difficult for the hole to recombine with the electron in the transfer process and eventually increase the transfer integral. But if the long-axis slip distance became too large, the promotion effect of the charge transfer which is obtained by the excellent electron and hole separation cannot overcome the disadvantages from the small overlap and eventually decrease the transfer integral. Therefore, appropriate molecular long-axis slip is favourable for hole transport, but the transfer integral will be greatly reduced if the slip is too large in *π* stacking.

**FIGURE 12:**
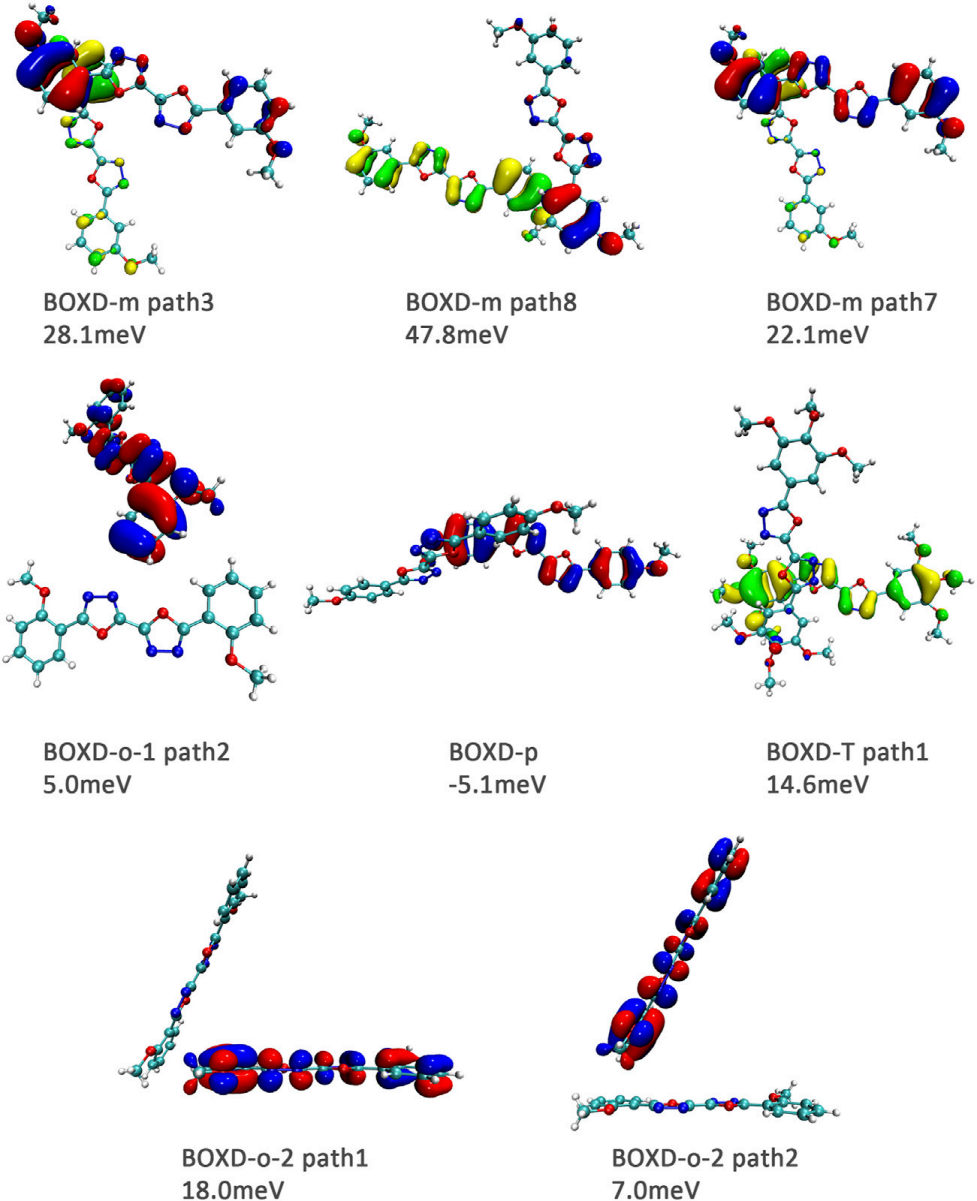
Bimolecular orbital distribution of primary hole transfer paths in herringbone arrangement. The positive phase is depicted in red and yellow, and the negative phase is depicted in blue and green.

Hole transfer integrals onto herringbone arrangement also show great difference from those in electron transport (Figure 12). Comparing the HOMOs of herringbone arrangement in BOXD-m, orbitals were distributed on two molecules and concentrated in the position of the molecular overlap. Just like those in *π* stacking, the transfer integral will be reduced due to the more concentrated orbital distribution and orbital overlap. While in BOXD-o-1, BOXD-p, and BOXD-T, the HOMOs are located in one of these pair of two molecules at the effect of Coulomb coupling. Due to the lack of the molecular orbital overlap, the transfer integral decreases obviously. At the same time, the increase in the dihedral angle has a negative effect on the transfer integral. Increasing the dihedral angle can also be seemed as another way to reduce the molecular overlap, making it difficult for hole transfer. Thus, dihedral angles can fundamentally affect the overlap of molecules and their orbitals to change the herringbone transfer integral. The difference of transfer integral between path 1 and path 2 in BOXD-o-2 can be found in hole mobility with the exactly opposite result. That is because of the distribution of HOMOs which is also quite opposite to that of LUMOs.

Orbital overlap integral of every primary hole transfer pathways is also evaluated (Table 3). The same with those in the electron transfer process, the reduced tendency of orbital overlap integral also can be found with the decrease in transfer integral (Figure S9 and Figure S10). In D–p2, p–p1 of *π* stacking and T–p1 of herringbone arrangement, larger orbital overlap integrals and smaller transfer integrals than o1–p1 and o2–p1 appear due to the disadvantage of molecular overlap.

**TABLE 3:** Transfer integral and orbital overlap integral of every primary hole pathways in *π* stacking and herringbone arrangement.

π stacking	m-p6	m-p2	m-p1	m-p5	o2-p3	D-p1	o1-p1	D-p2	p-p1	T-p2
Transfer integral	107.1	95	82.4	58.1	57.5	48.1	17.2	8.2	4.8	1.0
Orbital overlap	1.06E-02	9.20E-03	8.23E-03	7.53E-03	6.31E-03	5.30E-03	7.25E-04	2.15E-03	2.00E-03	3.71E-05
**Herringbone arrangement**	**m-p8**	**m-p3**	**m-p7**	**o2-p1**	**T-p1**	**o2-p2**	**p**	**o1-p2**	**-**	**-**
Transfer integral	47.8	28.1	22.1	18.0	14.6	7.0	5.1	5.0	-	-
Orbital overlap	5.11E-03	3.69E-03	2.93E-03	1.65E-03	1.97E-03	5.94E-04	7.11E-04	3.73E-04	-	-

## Conclusion

**FIGURE 13:**
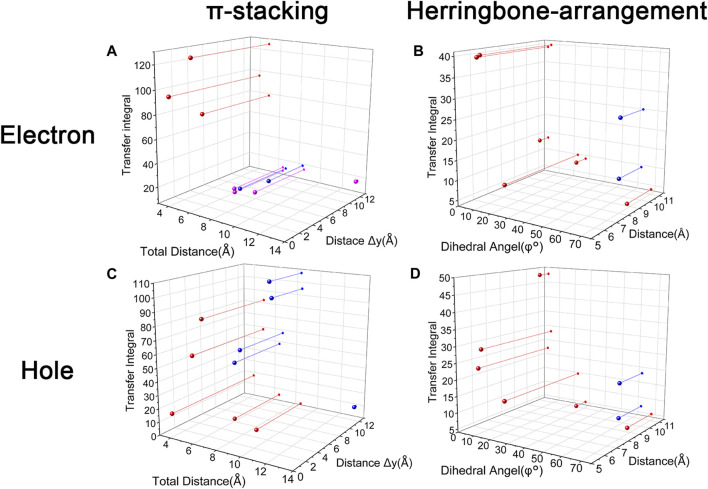
Relationship between transfer integral and crystal structure in stacking and herringbone arrangement. **(A)** Red: LUMOs distributed on both molecules with small slip distances. Pink: LUMOs distributed on both molecules with large slip distances. Blue: LUMOs distributed on one molecule. **(B)** Blue: The exception molecular of BOXD-o2. **(C)** Red: HOMOs are symmetrically distributed on each molecule. Blue: HOMOs are located on the overlap area. **(D)** Blue: The exception molecular of BOXD-o2.

Based on multiple model and high-precision first-principles computational analysis of dense packing of organic molecules, we finally reveal the effects of crystal structures with *π*-packing and herringbone arrangement for anisotropic electron and hole mobility. Intermolecular distances are the determining effect of transfer integral in *π* stacking. For the electron transfer process, the shorter intermolecular distance is better because the molecular orbital overlap is beneficial to the increase in transfer integral. While the overlap between the bonding and antibonding orbital greatly limits the integral when intermolecular distances become larger. Uneven distribution of molecular orbitals between molecules would also have a negative effect on this integral. However, the situation has difference in the hole transfer process. If the molecular orbitals are symmetrically distributed over each molecule, larger intermolecular distance will be detrimental to the transfer integral, which is same as electron transfer. But with the increase in the long axis critical slip distance, the transfer integral increases first and then decreases due to the separation of the electron and hole. The transfer integrals in herringbone arrangement which are usually smaller than those of *π* stacking are mainly controlled by the dihedral angle, except that the unique structure of BOXD-o-2 leads to its different transfer integrals. The transfer integral will decrease with the increase in the dihedral angle. According to Figure 13, small intermolecular distances, which are less than 6 Å, should be beneficial to charge transfer in *π* stacking, but it is also possible to achieve better mobility by appropriately increasing the distance in the hole transfer process. With regard to herringbone arrangement, the mobilities of parallel herringbone arrangement can even be comparable to that of *π* stacking; dihedral angles of more than 25° usually have extremely adverse effects on charge transfer. On the other hand, excessive structural relaxation also negatively impacted to attaining larger mobility. The almost nonexistent mobility of BOXD-T in hole transfer is ascribed to the combined influence of huge reorganization and small transfer integral. Actually, the different orientations of electron and hole mobilities in three dimensions can effectively inhibit or avoid carrier recombination. According to the results in Figure 4 and Figure 10, it can be noticed that except BOXD-p, the directions of maximum electron and hole transport are different in every crystalline phase, which can significantly reduce the possibility of carrier recombination. Based on the differences in their anisotropy of hole mobility in BOXD-m and BOXD-o1, their carrier recombination probabilities should slightly be higher than those in BOXD-o2, BOXD-D, and BOXD-T. This BOXD system can produce many completely different crystal structures simply by changing the position of the substituents. Through the systematic analysis of the structure–property relationship, the influence rule of intermolecular relative position and transfer integral as well as carrier mobility can be summarized. This relationship is based on the crystal structure and is applicable not only to the BOXD system but also to other molecular crystal systems. Our research plays an important role in theoretical explanation and prediction of charge mobilities and also makes a great contribution to control the anisotropy and enriches the material informatics. According to our research, people can better tailor the electron and hole materials more efficiently and more purposefully.

## Data Availability

The crystal structure data can be obtained free of charge *via* www.ccdc.cam.ac.uk/data_request/cif (CCDC numbers are 293679, 1448062, 1875779, and 1875781-1875783). Other data that support the findings of this study are available from the corresponding author on reasonable request.
