# Supplementary material for: Accurate Analysis of Anisotropic Carrier Mobility and Structure–property Relationships in Organic BOXD Crystalline Materials
Source: Front Chem. 2021 Nov 11;9:775747. doi: 10.3389/fchem.2021.775747 (PMC8631907; doi:10.3389/fchem.2021.775747)
Supplement: Supplementary file 1 [file Presentation1.pdf]

Accurate analysis of anisotropic carrier mobility and structure-property relationships in organic BOXD crystalline materials

Supplementary Material

Shi-Ping Wang[a], Yu Wang[a], Fang-Yi Chen[b], Hai-Tao Wang[b,*], Fu-Kit Sheong[c,*], Fu-Quan Bai[a,d,*] & Hong-Xing Zhang[a,*]

[a] Laboratory of Theoretical and Computational Chemistry, Institute of Theoretical Chemistry and College of Chemistry, Jilin University, Changchun, 130023, China.

[b] Key Laboratory of Automobile Materials (MOE), Institute of Materials Science and Engineering, Jilin University, Changchun, 130012, People's Republic of China.

[c] Department of Chemistry and Institute for Advanced Study, Hong Kong University of Science and Technology, Clear Water Bay, Hong Kong, China.

[d] Beijing National Laboratory for Molecular Sciences, Beijing 100013, China

*E-mail for H.-T. W.: haitao_wang@jlu.edu.cn

*E-mail for F.-K. S.: fksheong@connect.ust.hk

*E-mail for F.-Q.B.: baifq@jlu.edu.cn.

*E-mail for H.-X. Z.: zhanghx@jlu.edu.cn.

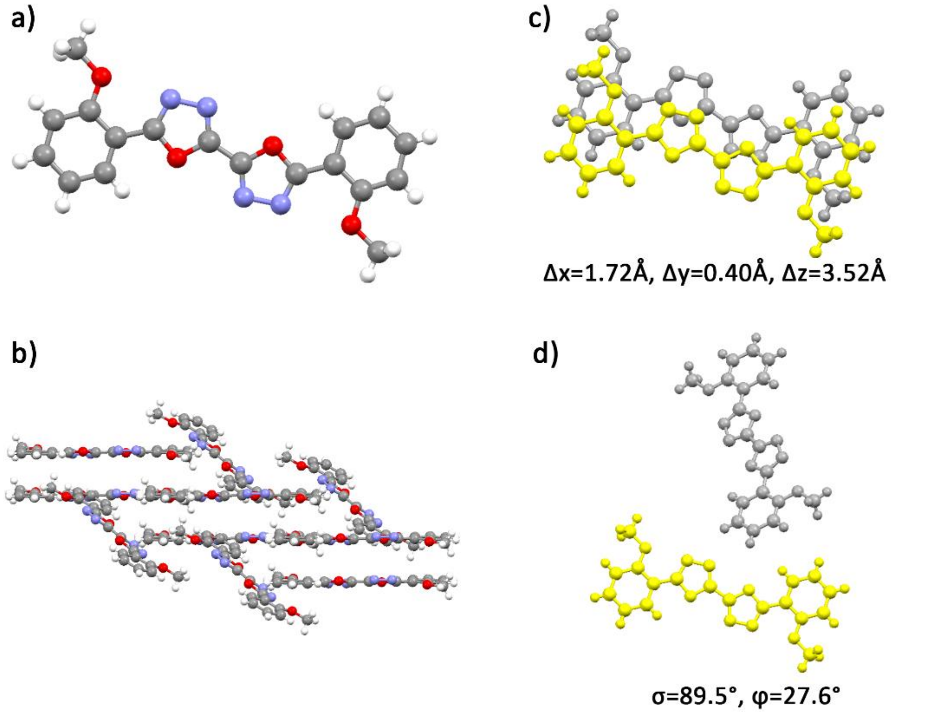

Figure S 1. a) Molecular structure of single molecular BOXD-o-1. b) Crystal structure of BOXD-o-1. c) Primary relative position of the bi-molecular in π-stacking. d) Primary relative position of the bi-molecular in herringbone-arrangement.

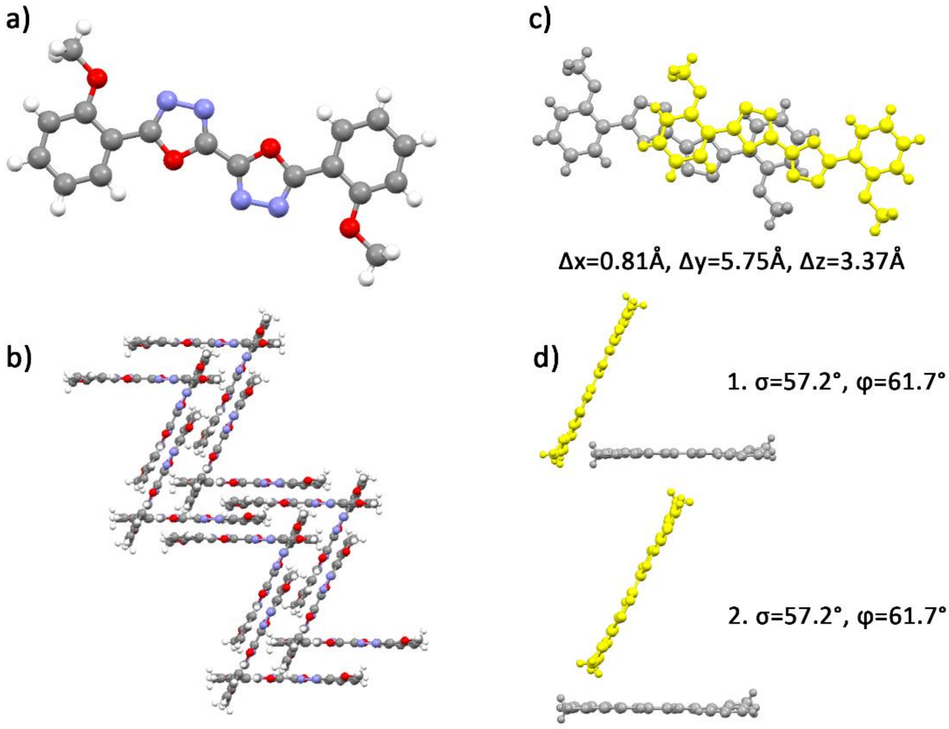

Figure S 2. a) Molecular structure of single molecular BOXD-o-2. b) Crystal structure of BOXD-o-2. c) Primary relative position of the bi-molecular in π-stacking. d) Primary relative position of the bi-molecular in herringbone-arrangement.

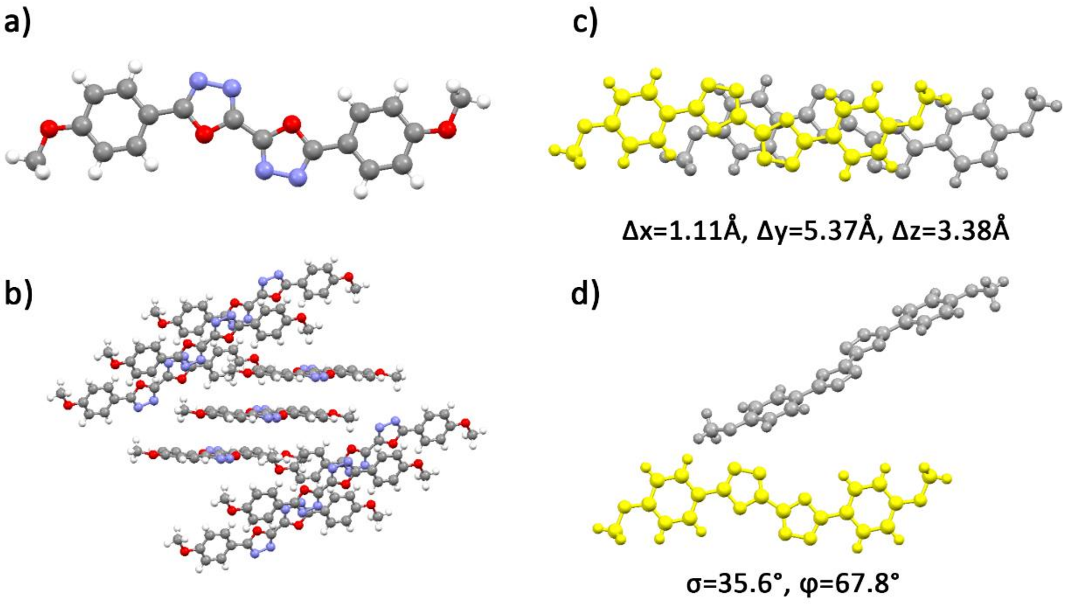

Figure S 3. a) Molecular structure of single molecular BOXD-p. b) Crystal structure of BOXD-p. c) Primary relative position of the bi-molecular in π-stacking. d) Primary relative position of the bi-molecular in herringbone-arrangement.

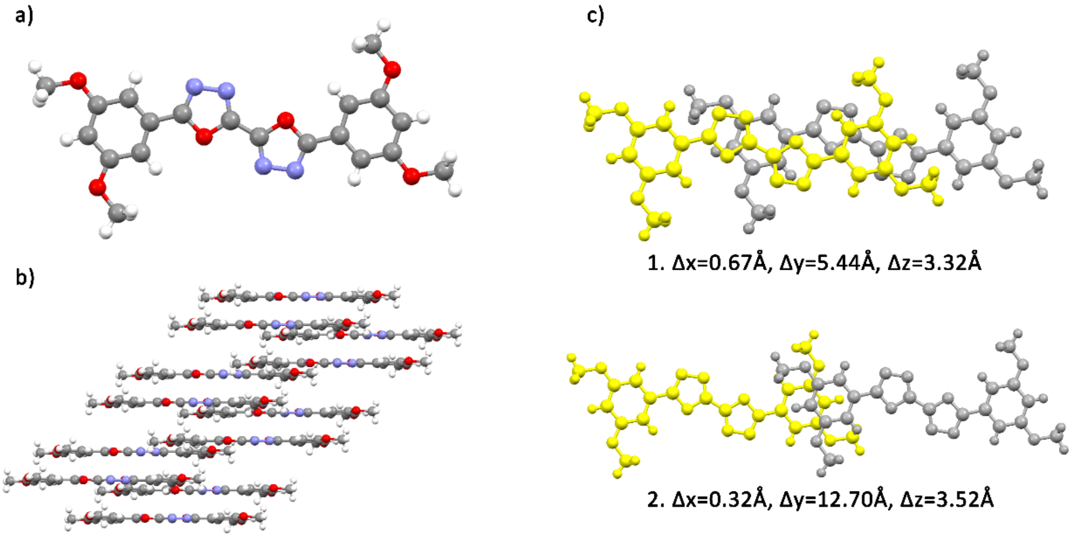

Figure S 4. a) Molecular structure of single molecular BOXD-D. b) Crystal structure of BOXD-D. c) Primary relative position of the bi-molecular in π-stacking.

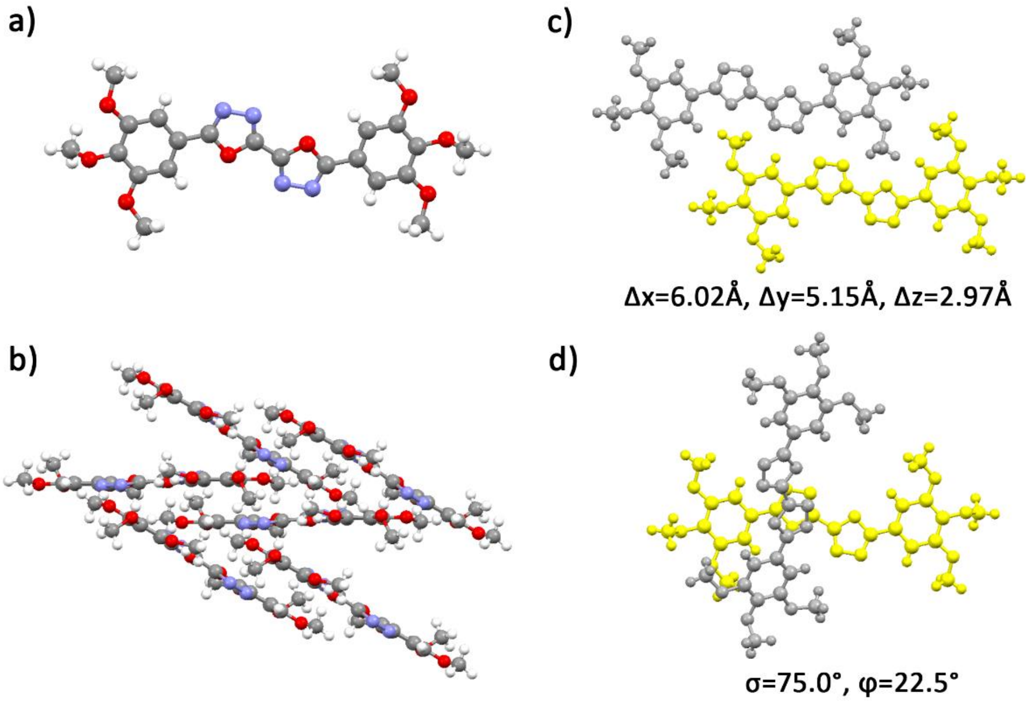

Figure S 5. a) Molecular structure of single molecular BOXD-T. b) Crystal structure of BOXD-T. c) Primary relative position of the bi-molecular in π-stacking. d) Primary relative position of the bi-molecular in herringbone-arrangement.

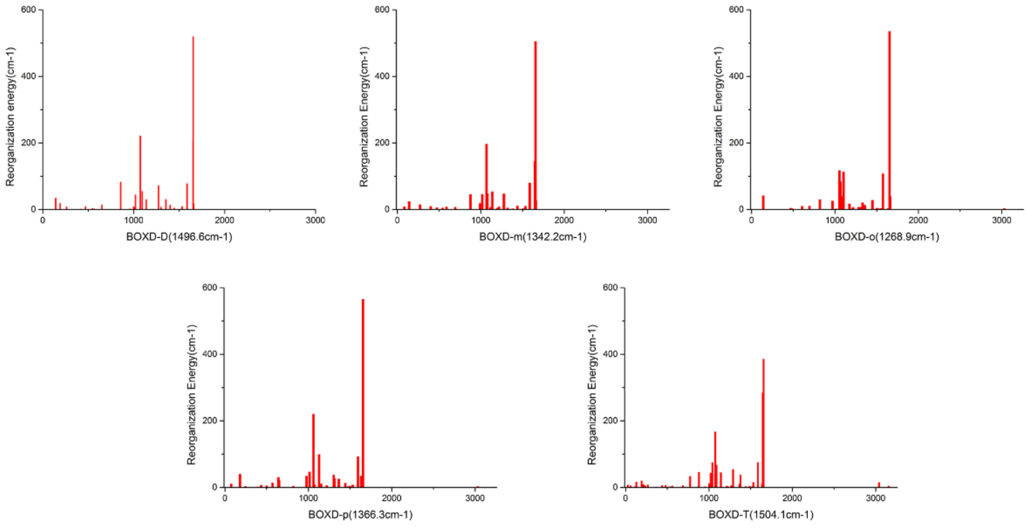

Figure S 6. Calculated reorganization energies of five molecular structure.

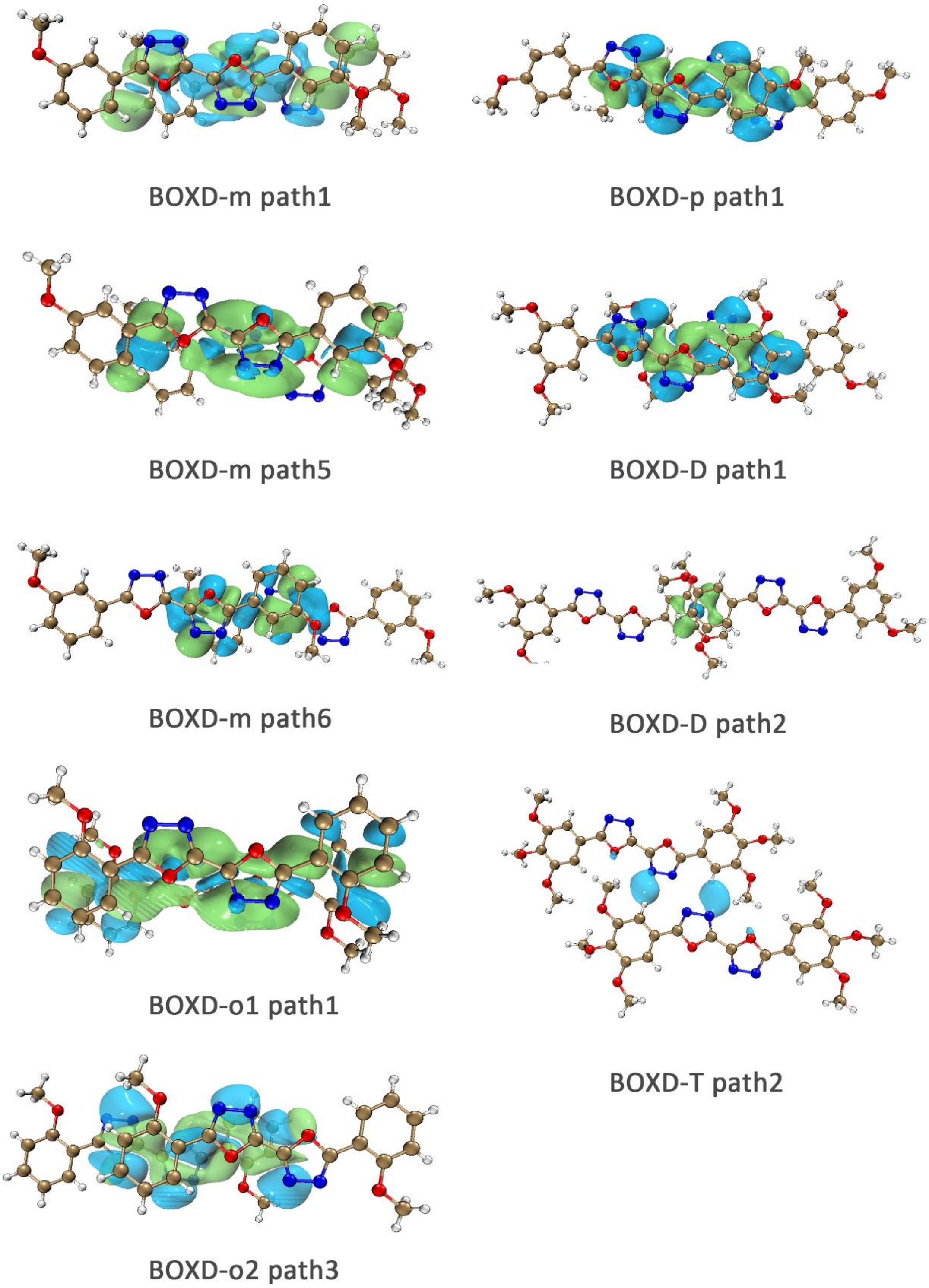

Figure S 7. The bi-molecular orbital overlap direction of primary electron transfer paths in π-stacking.

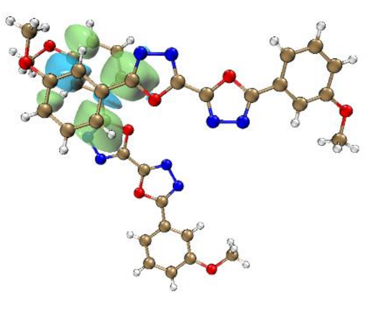

BOXD-m path3

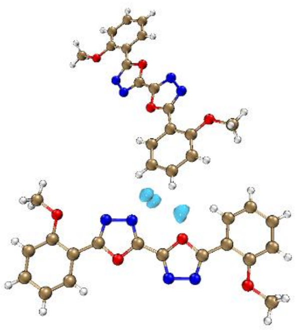

BOXD-o1 path2

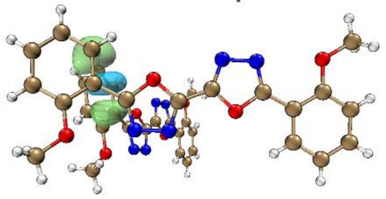

BOXD-o2 path1

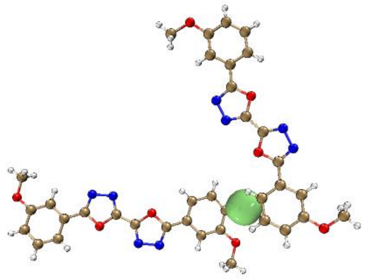

BOXD-m path4

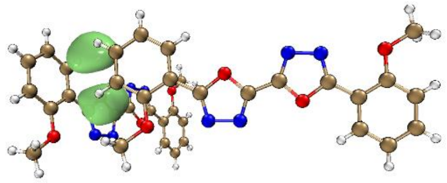

BOXD-o2 path2

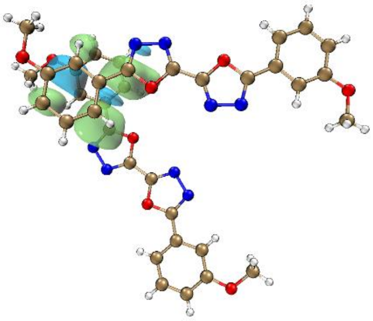

BOXD-m path7

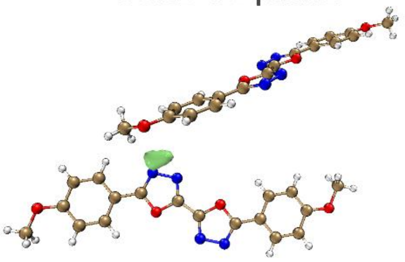

BOXD-p

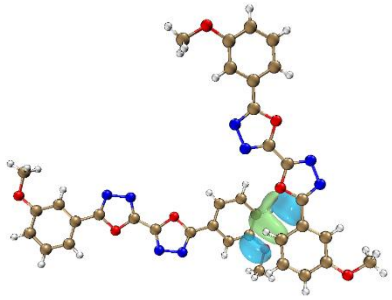

BOXD-m path8

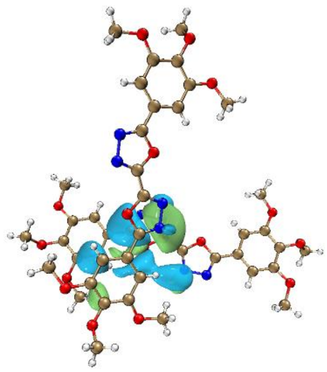

BOXD-T path1

Figure S 8. The bi-molecular orbital overlap direction of primary electron transfer paths in herringbone arrangement.

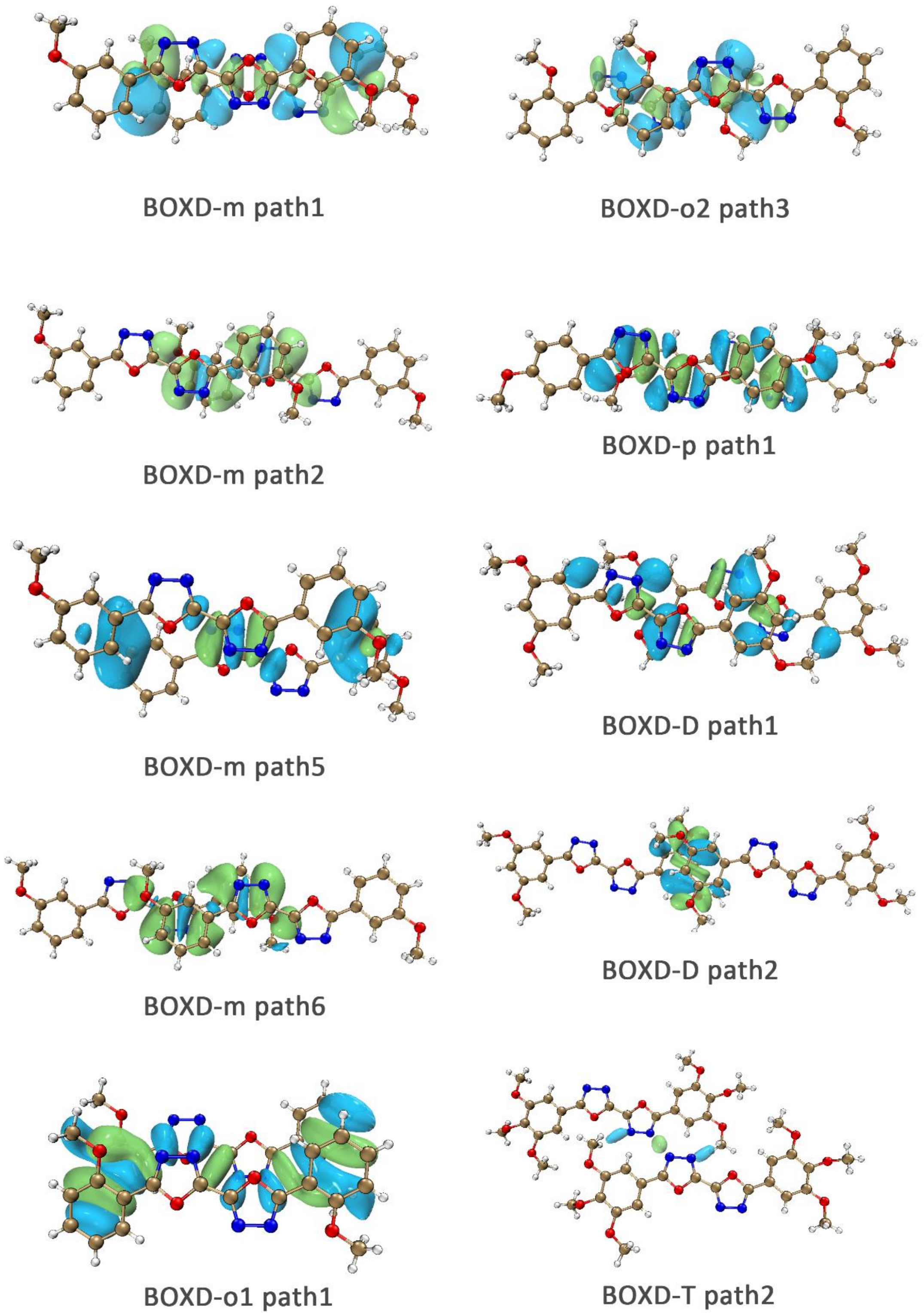

Figure S 9. The bi-molecular orbital overlap direction of primary hole transfer paths in π-stacking.

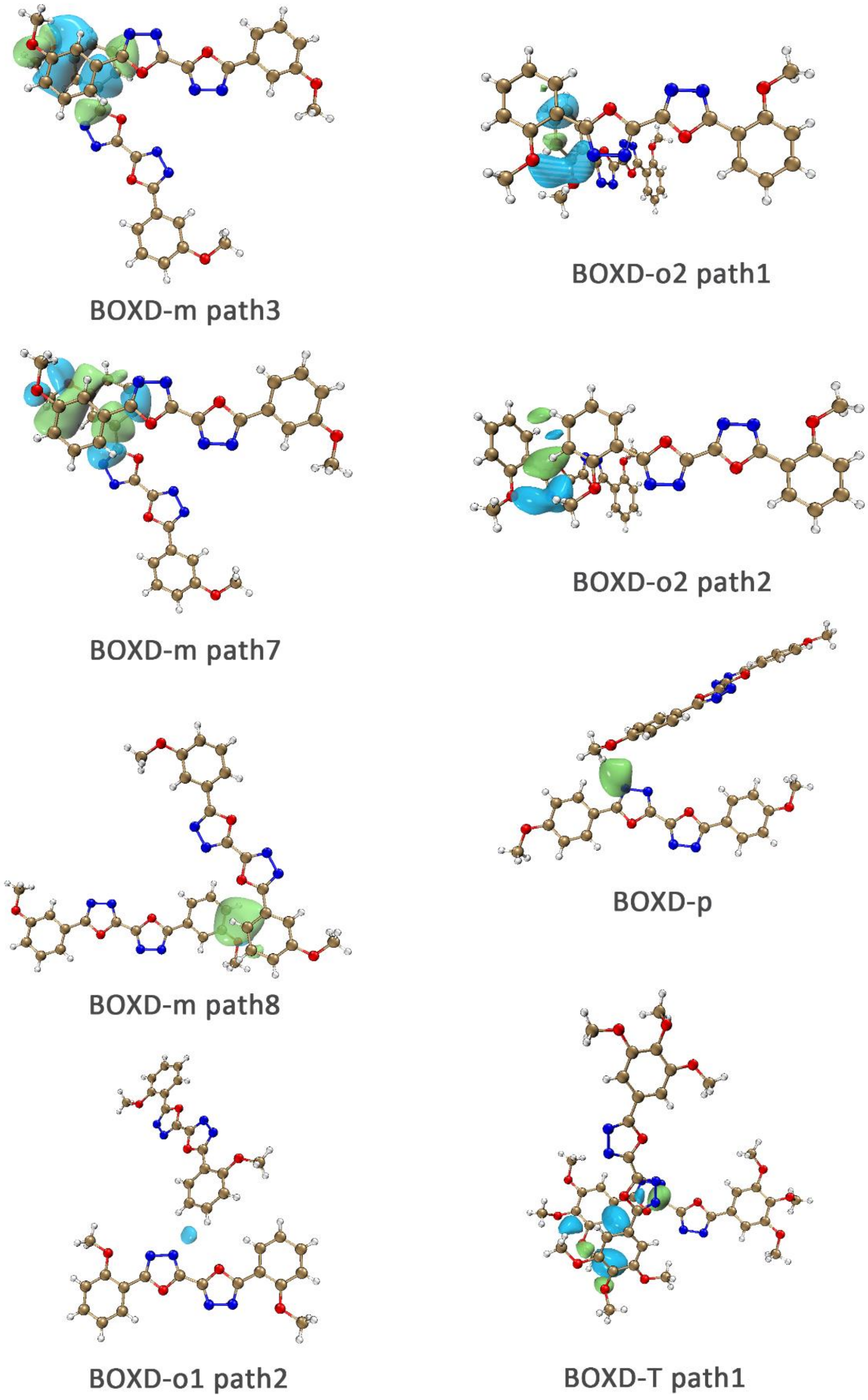

Figure S 10. The bi-molecular orbital overlap direction of primary hole transfer paths in herringbone arrangement.
